# Distinguishing *Sanghuangporus* from sanghuang-related fungi: a comparative and phylogenetic analysis based on mitogenomes

**DOI:** 10.1007/s00253-024-13207-1

**Published:** 2024-07-22

**Authors:** Xi-long Feng, Tian-chen Xie, Zhen-xin Wang, Chao Lin, Zhao-chen Li, Jinxi Huo, Yougui Li, Chengwei Liu, Jin-ming Gao, Jianzhao Qi

**Affiliations:** 1 Shaanxi Key Laboratory of Natural Products & Chemical Biology, College of Chemistry & Pharmacy, Northwest A&F University, Yangling, Xianyang, 712100 China; 2 Sericultural Research Institute, Zhejiang Academy of Agricultural Sciences, Hangzhou, 310021 China; 3 Key Laboratory for Enzyme and Enzyme-Like Material Engineering of Heilongjiang, College of Life Science, Northeast Forestry University, Harbin, 150040 China

**Keywords:** Comparative mitogenome, Phylogenetic analysis, Sanghuang-like fungi, *Sanghuangporus*

## Abstract

**Abstract:**

The Chinese medicinal fungi “Sanghuang” have been long recognized for their significant and valued medicinal properties, as documented in ancient medical literature. However, in traditional folk medicine, various macrofungi sharing similar appearance, habitat, and therapeutic effects with Sanghuang were erroneously used. These Sanghuang-like fungi mainly belong to the *Porodaedalea*, *Phellinus*, and *Inonotus* genera within the Hymenochaetaceae family. Despite the establishment of the *Sanghuangporus* genus and the identification of multiple species, the emerging taxonomic references based on morphological, ITS, and mycelial structural features have been inadequate to differentiate *Sanghuangporus* and Sanghuang-like fungi. To address this limitation, this study presents the first comparative and phylogenetic analysis of Sanghuang-related fungi based on mitogenomes. Our results show that *Sanghuangporus* species show marked convergence in mitochondrial genomic features and form a distinct monophyletic group based on phylogenetic analyses of five datasets. These results not only deepen our understanding of Sanghuang-like fungi but also offer novel insights into their mitochondrial composition and phylogeny, thereby providing new research tools for distinguishing members of the *Sanghuangporus* genus.

**Key points:**

*• Sanghuangporus* *,* *Inonotus, and Porodaedalea are monophyly in sanghuang-like species.*

*• Mitogenome-based analysis exhibits high resolution in sanghuang-like genus.*

*• The mitogenomes provide strong evidence for reclassifying Phellinus gilvus S12 as Sanghuangporus vaninii.*

**Supplementary Information:**

The online version contains supplementary material available at 10.1007/s00253-024-13207-1.

## Introduction

The esteemed macro-fungi known as “Sanghuang” have long been revered in traditional Chinese medicine for their therapeutic properties, with the earliest reference to “Sang’er” appearing in the ancient “Shen Nong Ben Cao Jing” (Yingjie and Wansheng 2016). The nomenclature “Sanghuang” was formalized in the Tang Dynasty’s “Treatise on Medicinal Properties (Yao Xing Lun)” (Zhen 2006), and it is recognized under various monikers, such as “Sanghwang” in South Korea and “Meshimakobu” in Japan. The Ming Dynasty’s comprehensive medical compendium, “Compendium of Materia Medica,” provides detailed accounts of its medicinal applications, predominantly in the regulation of bodily functions (Bao et al. 2013). However, the assignment of a Latin name to “Sanghuang” has been fraught with challenges due to the ambiguity surrounding synonyms and the misidentification of visually similar mushrooms. In folk medicine, certain *Phellinus* species have been erroneously considered as “Sanghuang” for their medicinal purposes. In more recent times, scholarly research has proposed *Inonotus hispidus* (Bull.) P. Karst. as the most plausible candidate for the “Sanghuang” documented in historical medical texts (Bao et al. 2013; Hai-ying et al. 2017). The discovery of *Inonotus sanghuang* Sheng H. Wu, T. Hatt. & Y.C. Dai marked a significant milestone, firmly linking the legendary “Sanghuang” to a tangible fungal entity. This species was identified through ITS sequencing and mycelial morphology, and it was observed to parasitise living mulberry trees (Shenghua et al. 2012). Consequently, the genus *Sanghuangporus* was established as a distinct taxon (Zhou et al. 2016), classified within the Hymenochaetaceae family of the Agaricomycetes. The Latin designation for “Sanghuang” was thus confirmed as *Sanghuangporus sanghuang*. *Sanghuangporus* mushrooms are endemic to a variety of global regions, including China, the Philippines, Australia, Japan, Korea, and North America, with a total of 14 known species (Sheng-Hua and Yu-Cheng 2020; Shenghua et al. 2012; Wu et al. 2019). Notably, these fungi exhibit a preference for specific tree species; for instance, *S. sanghuang* is exclusively associated with mulberry trees. Nonetheless, fungi resembling *Sanghuangporus* (Zhang et al. 2022), such as those from the genera *Phellinus*, *Inonotus*, and *Fomitiporia*, pose a challenge in differentiation based solely on morphology. The absence of a standardized identification protocol for *Sanghuangporus* species presents a formidable barrier to research, commerce, and the development of these mushrooms, thereby limiting their potential applications and industrial use.

In the realm of traditional Chinese medicine, “Sanghuang” is revered alongside other esteemed medicinal fungi, such as *Ganoderma lucidum* (Lingzhi), *Hericium erinaceus*, *Wolfiporia cocos*, and *Taiwanofungus camphoratus*, owing to their extensive medicinal histories (Zhou et al. 2022). Recent pharmacological investigations have uncovered a range of potent biological activities in the extracts of Sanghuangporus mushrooms, including antioxidant (Cai et al. 2019; Lin et al. 2017b; Liu et al. 2017; Ma et al. 2022), anti-inflammatory (Lin et al. 2017a), immunomodulatory (Yin et al. 2022), anti-tumor (Cheng et al. 2022a, b; He et al. 2021b; Wu et al. 2023), anti-diabetic (Huang et al. 2022a, b), antiviral (Chien et al. 2022), and therapeutic effects against gouty arthritis and hyperuricemia (Song et al. 2023; Sun et al. 2022). The monomeric compounds derived from Sanghuangporus mushrooms, such as triterpenoids and polyphenols, mirror these pharmacological activities (Cheng et al. 2019; Chepkirui et al. 2018; Jin-Jin et al. 2021). Intriguingly, the crude extracts, monomeric compounds, and their pharmacological activities of Sanghuangporus mushrooms are akin to those of Sanghuang-like fungi (He et al. 2021a; Lee and Yun 2011; Wang et al. 2022; Yan et al. 2017). This similarity in chemical composition and pharmacological efficacy may account for the historical difficulty in distinguishing between these fungi. While the ITS region remains the prevalent method for species identification (Seifert et al. 2007), it offers limited phylogenetic resolution and fails to capture the genetic relationships among closely related fungal taxa, such as Sanghuangporus and Sanghuang-like mushrooms. In contrast, mitochondrial DNA (mtDNA), inherited uniparentally and without recombination, exhibits high conservation in its coding regions and rapid mutation in its non-coding regions, leading to diverse DNA polymorphisms (Basse 2010; Xu et al. 2009). Utilizing mtDNA as a molecular marker can thus illuminate species relationships at the molecular level, overcoming the limitations of traditional morphology-based fungal taxonomy. Therefore, comparative genomic and phylogenetic analyses based on mitogenomes offer an effective strategy to differentiate between Sanghuangporus and Sanghuang-like mushrooms.

Given the mitogenome’s pivotal role in a myriad of physiological and biochemical processes, such as cell growth, development, stress resistance, energy metabolism, senescence, and apoptosis (Gray et al. 2001; Lang et al. 1999; Latorre-Pellicer et al. 2016), it is often regarded as the “second genome” of eukaryotes (Jørgensen et al. 2018). Mitogenome studies have significantly advanced the taxonomy, phylogeny, and evolutionary analysis of insects (Bae et al. 2004; Li and Li 2022; Li et al. 2023a; Nardi et al. 2003). In fungi, an increasing number of mitogenomes have been sequenced, facilitating the study of phytopathogenic fungi (Li et al. 2023b; Yildiz and Ozkilinc 2021), elucidating the genetic, evolutionary, and environmental adaptations of ectomycorrhizal fungi (Li et al. 2020a, 2019, b, 2021, c), and revealing the population characteristics of Agaricaceae mushrooms (Araujo et al. 2021). Although partial comparative and phylogenetic analyses based on the nuclear genome of *Sanghuangporus* and Sanghuang-like mushrooms have been reported (Wu et al. 2019; Zhang et al. 2022; Zhou et al. 2016; Zhu et al. 2019), no studies have employed mitogenome-based comparative and phylogenetic analyses to distinguish these fungi. In this study, we present the characterization of six mitogenomes from Sanghuang-like mushrooms for the first time and conduct a comprehensive comparative and phylogenetic analysis of the mitogenomes of *Sanghuangporus* and Sanghuang-like mushrooms within the order Hymenochaetales. Our findings enhance the understanding of the evolutionary origins, genetic diversity, and phylogenetic relationships of these significant medicinal mushrooms and provide novel molecular insights to distinguish between *Sanghuangporus* and Sanghuang-like fungi.

Since mitogenome is involved in various physiological and biochemical processes, such as cell growth and development, stress resistance, energy metabolism, aging, and apoptosis (Gray et al. 2001; Lang et al. 1999; Latorre-Pellicer et al. 2016), it is considered the “second genome” of eukaryotes (Jørgensen et al. 2018). The studies of mitogenomes greatly contributed to the taxonomic, phylogenetic, and evolutionary analysis of insects (Bae et al. 2004; Li and Li 2022; Li et al. 2023a; Nardi et al. 2003). In recent years, an increasing number of mitogenomes of fungi have been reported that have been used to study phytopathogenic fungi (Li et al. 2023b; Yildiz and Ozkilinc 2021); to characterize genetics, evolution, and environmental adaptations of the ectomycorrhizal fungi (Li et al. 2020a, 2019, b, 2021, c); and to reveal the population characteristics of the mitogenomes of the Agaricaceae mushrooms (Araujo et al. 2021). Although comparative and phylogenetic analyses based on the nuclear genome of *Sanghuangporus* and Sanghuang-like mushrooms have been reported partially (Wu et al. 2019; Zhang et al. 2022; Zhou et al. 2016; Zhu et al. 2019), no studies have reported using comparative and phylogenetic analyses based on the mitogenome to distinguish them. In this study, we characterized six mitogenomes of Sanghuang-like mushrooms for the first time and conducted comparative and phylogenetic analyses of the mitogenomes of *Sanghuangporus* and Sanghuang-like mushrooms within the order Hymenochaetales. These findings promote the understanding of the origin, evolution, genetic diversity, and phylogeny of these important medicinal mushrooms and provide new molecular evidence to distinguish between *Sanghuangporus* and Sanghuang-like mushrooms.

## Material and methods

### Fungal isolates origin, DNA extraction, and sequencing

The wild fruiting bodies of *Inonotus hispidus* (Zhang et al. 2022) was collected in Xinjiang Province, China, and then stored in Shaanxi Province Key Laboratory of Chemical Biology & Natural Products. The wild growing fruiting body of *Phellinus gilvus* S12(Huo et al. 2020) was isolated from a mulberry tree in Zhejiang Province, China. The two strains were cultured in PDB medium (200 rpm, 25 °C) for 1 week to obtain mycelia. The mycelia were collected by centrifugation, rinsed twice with sterile water, and then centrifuged to remove water. Genomic DNA was isolated using the sodium dodecyl sulfate technique after the mycelium was crushed with liquid nitrogen and integrity checked by agarose gel electrophoresis. Raw sequencing data for *Phellinus viticola*, *Porodaedalea chrysoloma*, *Phellinus ferrugineofuscus*, and *Porodaedalea niemelaei* were obtained in JGI database*.*

The whole-genome sequencing was performed using a combined strategy of Illumina NovaSeq and Nanopore sequencing technology and obtained 230-fold average genome coverage, with a paired-end library. Then, the reads were de novo assembled by SOAPdenovo 2.0425(Li et al. 2015). The sequencing data from Nanopore platform were corrected according to mapping the Illumina sequencing reads by BLASR26(Chaisson and Tesler 2012), and then assembled by the CeleraAssembler (Myers et al. 2000) After reliable scaffolds were generated, the correction of the sequencing reads was performed again based on the Illumina data. The final polished assemblies represent the complete genome sequences.

### New mitogenomes assembly and annotation

The mitogenomes of *I. hispidus* and *P. gilvus* were de novo assembled from Nanopore raw reads using minimap2 v2.17-r94 (Li 2018) and miniasm v0.3-r179(Li 2016), and further refined using racon v1.4.20 (Vaser et al. 2017) and pilon v1.23(Walker et al. 2014), based on Illumina data. The final assemblies were assessed for quality using samtools (Danecek et al. 2021).

The mitogenomes were then annotated using the MFannot online software (Lang et al. 2023) with the genetic code 4 for predicting protein-coding genes (PCGs), tRNA genes, rRNA genes, and partial open reading frames. The annotation was manually proofread, and the tRNA and rRNA genes were further verified using RNAweasel (Lang et al. 2007) and tRNAScan (Lowe and Chan 2016), respectively. The type I intron was checked for its conformance to normal sequence characteristics using RNAweasel. The starting and ending positions of *rns*, *rps3*, the 14 conserved PCGs, and intron insertion sites were verified using MAFFT (Katoh et al. 2002) and NCBI blast analysis. ORF Finder (Rombel et al. 2002) was used to search for open reading frames in intergenic regions and intron regions longer than 300 bp, and the starting points and functions of ORFs in the intron were determined using Blastn and Blastp. Finally, the graphical maps of the complete mitogenomes were drawn using OGDraw v1.2(Greiner et al. 2019).

### Sequence analysis of mitogenomes

We conducted a comprehensive analysis of the mitochondrial genomes of 16 species, including basic composition, relative synonymous codon usage, and selective pressure. We calculated the GC content = ((G + C)/(G + C + A + T)), GC skew = ((G − C)/(G + C)), and AT skew = ((A − T)/ (A + T)) of core genes, intronic regions, uORFs, and non-coding regions in mitochondria. The relative synonymous codon usage (RSCU) values of concatenated sequences of mitochondrial core and non-core genes were clustered using PCA, and the usage and expression levels of codons encoding different amino acids were presented using bubble plots. We also calculated the synonymous (Ks) and nonsynonymous (Ka) substitution rates via DnaSPv6.12.03 (Rozas et al. 2017) of 15 conserved coding genes (*atp6*, *atp8*, *atp9*, *cob*, *cox1*, *cox2*, *cox3*, *nad1*, *nad2*, *nad3*, *nad4*, *nad4l*, *nad5*, *nad6*, and *rps3*) and determined the Ka/Ks ratio to investigate selective pressure. The genetic distance of conserved PCGs was analyzed using MEGA 11 (Tamura et al. 2021) with the Kimura-2-parameter (K2P) substitution model. Tandem repeats were identified using Tandem Repeat Finder v4.07b(Benson 1999).

To compare the mitochondrial genomes of 16 species, various features were analyzed, including genome length, GC content, intron numbers, coding gene length, intronic region length, uORFs length, and core gene arrangement. These data were visualized using a stacked bar chart. To analyze synteny at the whole genome level, AliTV (Ankenbrand et al. 2017) was employed, starting from *cox1*. In order to classify mitochondrial gene introns in the 16 species, including those in *cox1*, different position classes (Pcls) were classified. Introns inserted at the same position of the *cox1* reference gene belonging to the same Pcl were named according to their insertion position, and the same Pcls usually have high sequence similarity.

### Phylogenetic inference

To investigate the evolutionary position in the phylum Basidiomycota, we first collected various mitochondrial genomes and finally constructed phylogenetic trees using amino acids of the 14 conserved PCGs of 103 Basidiomycete species. And then, we constructed four datasets based on 14 conserved PCGs, the genes for ribosomal large and small subunit (*rnl* and *rns*) of 16 Sanghuang-related species, including two *Sanghuangporus* and 14 Sanghuang-like fungi. They are (1) PCG: concatenated sequences of 14 conserved PCGs; (2) PCG12: combined 1st and 2nd codon positions of 14 conserved genes; (3) PCGR: concatenated 14 conserved PCGs and rRNA; (4) PCG12R: combined 1st and 2nd codon positions of 14 conserved genes and rRNA.

We first extracted the amino acid transcripts of the 14 conserved PCGs based on the standard translate codon code 4 for all 103 species for the AA dataset and coding sequences of the 14 conserved for other four datasets respectively with Snapgene viewer (v6.0.2). MAFFT (v7.453) was used to align the individual transcripts or coding gens, with default parameters. After using Gblocks and Trimal to eliminate the poorly aligned blocks, we used Phylosuite (v1.2.2) (Zhang et al. 2020) to concatenate the distributed protein sequences into a combined transcript set and coding genes sequences into combined sequences. Partition Finder2.1.1 (Lanfear et al. 2017) was used to determine the best partitioning scheme and evolutionary models for the combined protein sequence set and other four coding gene sequence sets, with greedy algorithm and AICc criterion. After analyzing the base substitution saturation and heterogeneity of sequences in amino acid transcripts and the four datasets by AliGROOVE v1.07 (Kück et al. 2014) and DAMBE (Xia 2018), phylogenetic trees were constructed with Bayesian inference (BI) and maximum likelihood (ML) methods, respectively, performed by MrBayes (v3.2.7) (Huelsenbeck and Ronquist 2001) and IQ-TREE (v1.6.8) (Minh et al. 2020). For the BI method, two runs with 4 chains, 2 × 10^6^ generations with sampling frequency set to 100 and burn-in fraction set to 25%. We assumed that iterations had reached steady state when estimated Bayesian posterior probabilities (BPP) were close to 1 and bootstrap (BS) = 100. IQ-TREE was used to conduct the ML method under the LG + R10 + F model for 5,000 bootstrap replicates, as well as the Shimodaira–Hasegawa–like approximate likelihood-ratio test. The final phylogenetic tree was visualized using FigTree v1.4.4.

## Results

### Features of the six Sanghuang-like mitogenomes

**Fig. 1:**
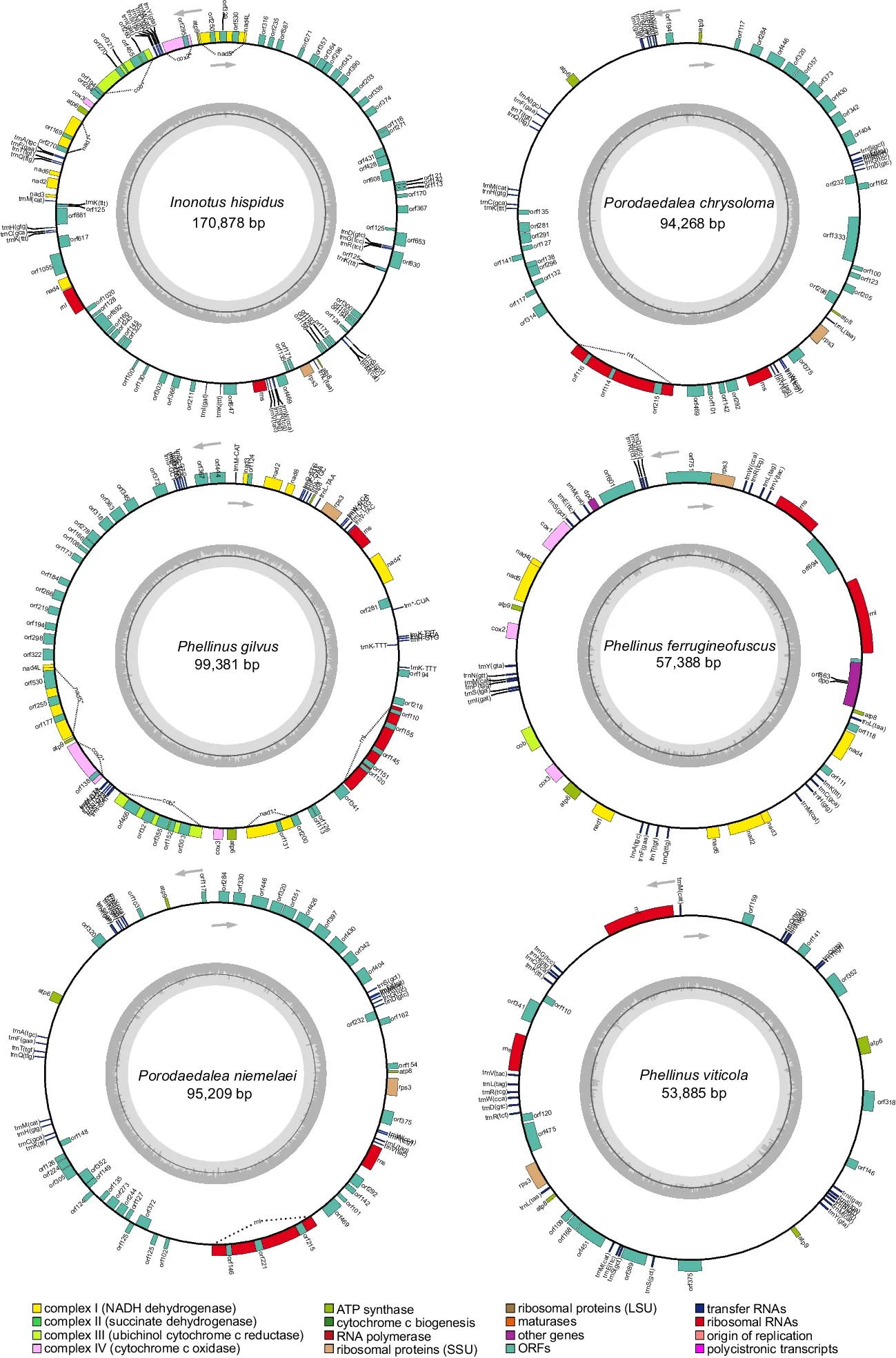
Circular mitogenomic maps of six Sanghuang-like fungi, *Inonotus hispidus*, *Phellinus gilvus*, *Phellinus viticola*, *Phellinus ferrugineofuscus*, *Porodaedalea niemelaei*, and *Porodaedalae chrysoloma*. Genes with certain functions are displayed in different colored boxes. The arrow indicates the direction of the gene from start to end

The complete mitogenomes of six Sanghuang-like species, *Inonotus hispidus*, *Phellinus gilvus*, *Phellinus viticola*, *Porodaedalea chrysoloma*, *Phellinus ferrugineofuscus*, and *Porodaedalea niemelaei*, were all composed of circular DNA molecules. The sizes of the six mitogenomes varied significantly, with a range from 53, 885 bp (*P. viticola*) to 170, 878 bp (*I. hispidus*), representing up to a threefold difference in size (Fig. 1). All six fungi possessed a typical set of 14 core protein-coding genes (PCGs) involved in energy metabolism, a single *rps3* gene involved in translation, and two rRNA genes (*rnl* and *rns*), along with varying amounts of tRNA genes (Fig. 1, Table S1). The number of tRNAs in the six mitogenomes ranged from 24 (*P. niemelaei*) to 29 (*I. hispidus*) (Fig. S1-S6). Among the 29 tRNAs in *I. hispidus*, there are 4 copies of *trnK* responsible for the transport of lysine, and their anticodons are all UUU. The *trnR* responsible for arginine has two copies, but it has two anticodons, UCU and UCG (Tables S2). These tRNAs encoded all 20 amino acids and were found to range in size from 70 to 89 nucleotides, as outlined in Table S2.

### Characterization of the Sanghuang-related fungi mitogenomes

**Fig. 2:**
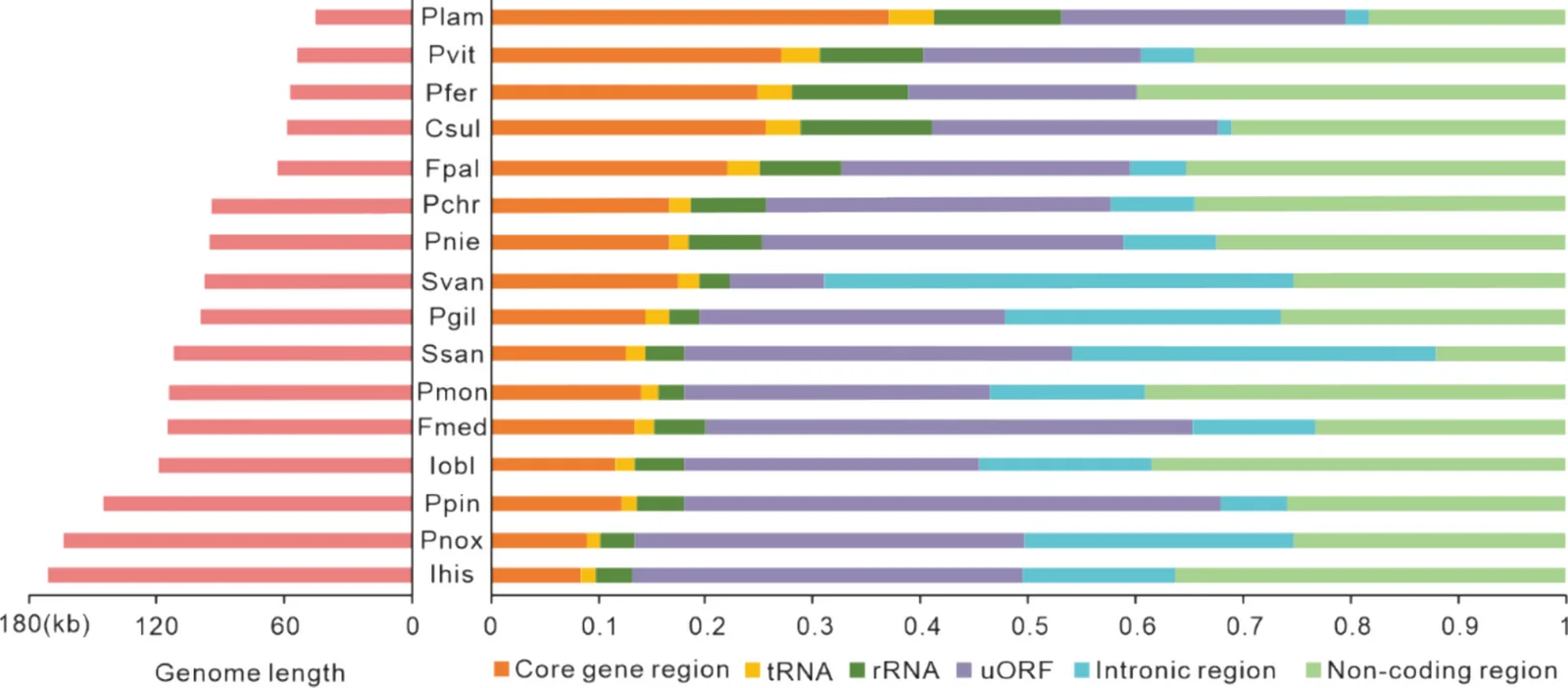
Mitochondrial relative composition and genome size. The relative composition of intronic region, uORFs, protein-coding region, intergenic region, and RNA gene region is shown in percentage, while the mitochondrial genome total size is displayed in base pairs

A total of 16 Sanghuang-related fungi mitogenomes (Table S3), including six Sanghuang-like fungi assembled and annotated by us, as well as the reported two *Sanghuangporus*, and eight Sanghuang-like (Fig. S7), were used for comparative analysis. These mitogenomes varied significantly at both inter and intraspecific levels (Fig. 2), ranging from 45,604 bp (*Phellinus lamaoensis*) to 170,878 bp (*Inonotus hispidus*) in size, with length differences of up to threefold. In terms of mitogenome composition, the intergenic regions, including intronic region and non-coding region, were the most abundant, accounting for an average of 43.68% of 16 mitogenomes. In contrast, RNA regions contributed the least, on average 8.37%, to the length of the whole mitogenomes (Fig. 2, and Table S3). The highly size-conserved core gene region, ranging from 13,854 bp (*I. obliquus*) to 17,754 bp (*P. pini*) makes its proportion in the mitogenome inversely correlated with the size of the mitogenome itself (Fig. 2). The mitogenome usually contains a set of uORFs that encode unknown proteins or known proteins without correct identification, GIY endonuclease and LAGLIDADG endonuclease are the only two members of uORFs known (Burger et al. 2012). With the exception of *S. vaninii*, uORFs and non-coding regions are the main factors contributing to interspecific differences in mitogenome size (Fig. 2).

Further mitogenomic content analysis revealed several noteworthy findings in Sanghuang-related fungi. *Porodaedalea. pini* possesses the most uORFs (100), with 15 GIY and LAGLIDADG endonucleases, whereas *I. hispidus* holds the most GIY and LAGLIDADG endonucleases (22), in 67 uORFs. Two rRNAs (*rns* and *rnl*) were found in all mitogenomes. The mitogenomes contained 24–30 tRNAs, indicating similar capacity to carry amino acids. In contrast to the quantitative conservation of tRNAs, GC content highlighted interspecific variation, ranging from 23.21 to 34.55%, but most did not exceed 30%. *S. vaninii* and *P. gilvus* have similarly high GC skews of 7.44% and 7.27%, respectively, while the GC skews of other mitogenomes were not greater than 5.43%. Furthermore, they possess the very similar positive AT skews, whereas most of the others have negative AT skews. The high similarity of the sequence features of *S. vaninii* and *P. gilvus* suggests that they have a close phylogenetic relationship (Table S3-S4).

### Repetitive sequence and tandem repeats

A total of seven repetitive sequences were detected and identified in the mitogenome of *S. sanghuang*, three in that of *S. vaninii*, and eight in that of *P. gilvus*, by comparison of the whole mitogenomes against themselves via BLASTn analysis. The size of repetitive sequences in the 16 Sanghuang-related mitogenomes ranged from 35 to 3422 bp, among which *I. hispidus* contained the largest and the second largest repeat sequences (2422 bp and 2428 bp). The third largest repeat sequence (1809 bp) was identified in *I. obliquus.* The range of pairwise nucleotide identities among the three mitogenomes was 77.26 to 100%. The repetitive sequences accounted for 1.76% to 1.86% of the three mitogenomes. The highest proportion of repeat sequences was found in *S. sanghuang*, while *S. vaninii* had the lowest content of repetitive sequences (Table S5).

Totals of 40, 19, and 15 tandem repeats were detected in the mitogenomes of *S. sanghuang*, *S. vaninii*, and *P. gilvus*, respectively. The longest tandem repeat sequence of 102 bp was identified in *I. obliquus*. Most of the tandem repeats were duplicated once or twice among the mitogenomes, with the highest number of duplications (28) in the *P. viticola* mitogenome. The proportion of tandem repeat sequences gradually decreased in the mitogenomes of *S. sanghuang*, *S. vaninii*, and *P. gilvus*, which were 1.81%, 0.67%, and 0.95%, respectively (Table S6).

### Variation, genetic distance, and evolutionary rates of core genes

**Fig. 3:**
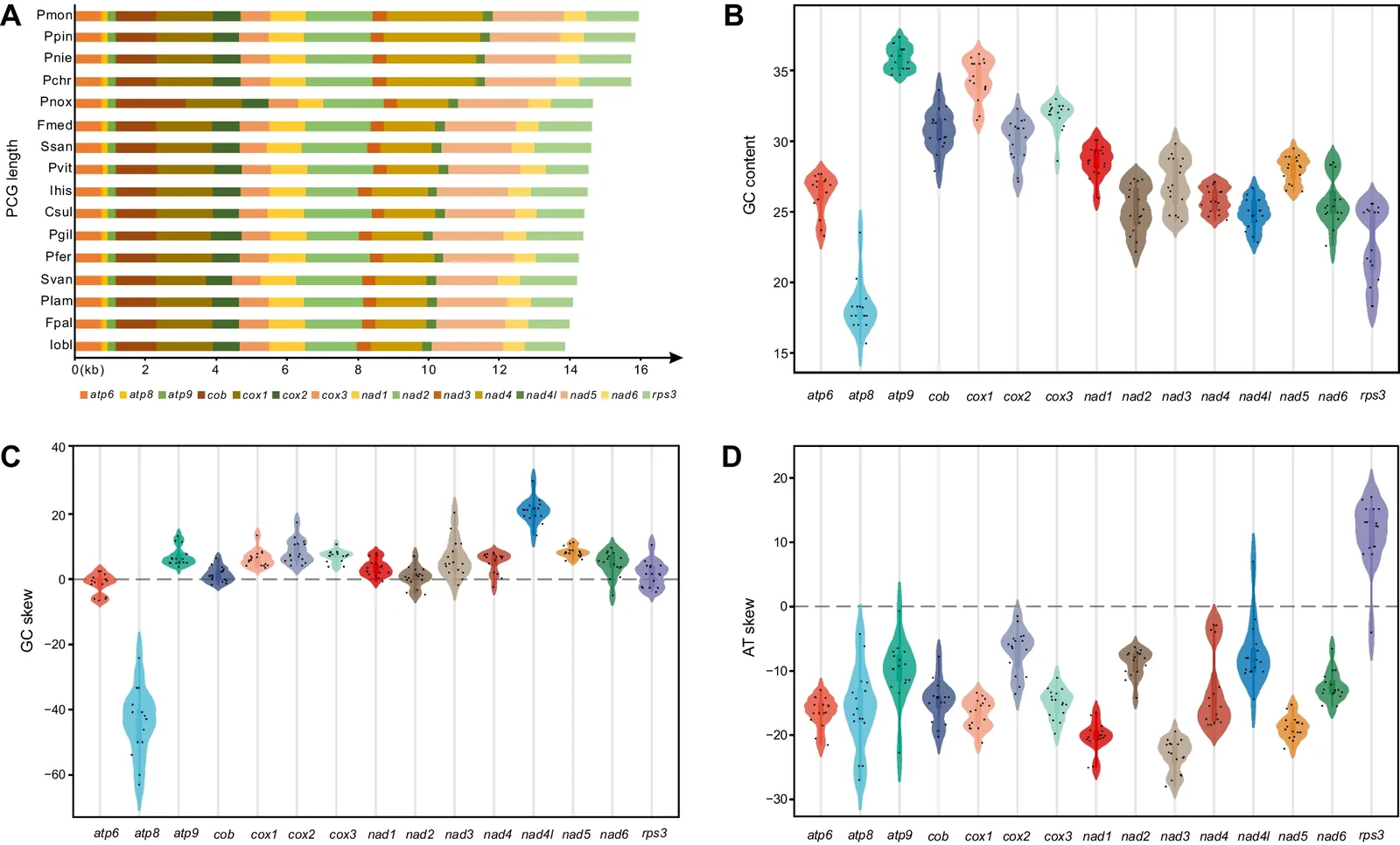
Variation in the length and base composition based on 14 conserved protein-coding genes and ribosomal protein (*rps3*) of Sanghuang-related species. **A** Length variation; **B** GC content; **C** GC skew; **D** AT skew of the 15 PCGs

Statistical analysis based on the conserved PCG and *rps3* reflects interesting common features of the Sanghuang-related mushrooms. Length-based linear alignment of 14 PCGs and *rps3* from 16 species showed that *nad4* showed conservation within the genus *Porodaedalea*, with a slightly longer length than those of other Sanghuang-like fungi outside the genus. The *cob* of *P. noxius* showed an unusual excess length compared to other species. Otherwise, each class of genes showed high conservation (Fig. 3A).

Most of PCGs exhibited negative AT (− 0.125 on average) and positive GC (0.024 on average) skew among the 16 species with high AT content (Table S7). The GC content and GC skew of *atp8* were 18.2% and 43.9%, respectively, much lower than those of other genes, which suggest that *atp8* could act as a marker for the start of mitochondrial gene replication with the lagging strand (Fig. 3B, C). The *rps3* had the highest AT skew values and the values were extensively distributed, ranging from − 4.04 to 17.04%, which implies low GC content and a high mutation rate in *rps3* within a low Tm (Fig. 3D).

**Fig. 4:**
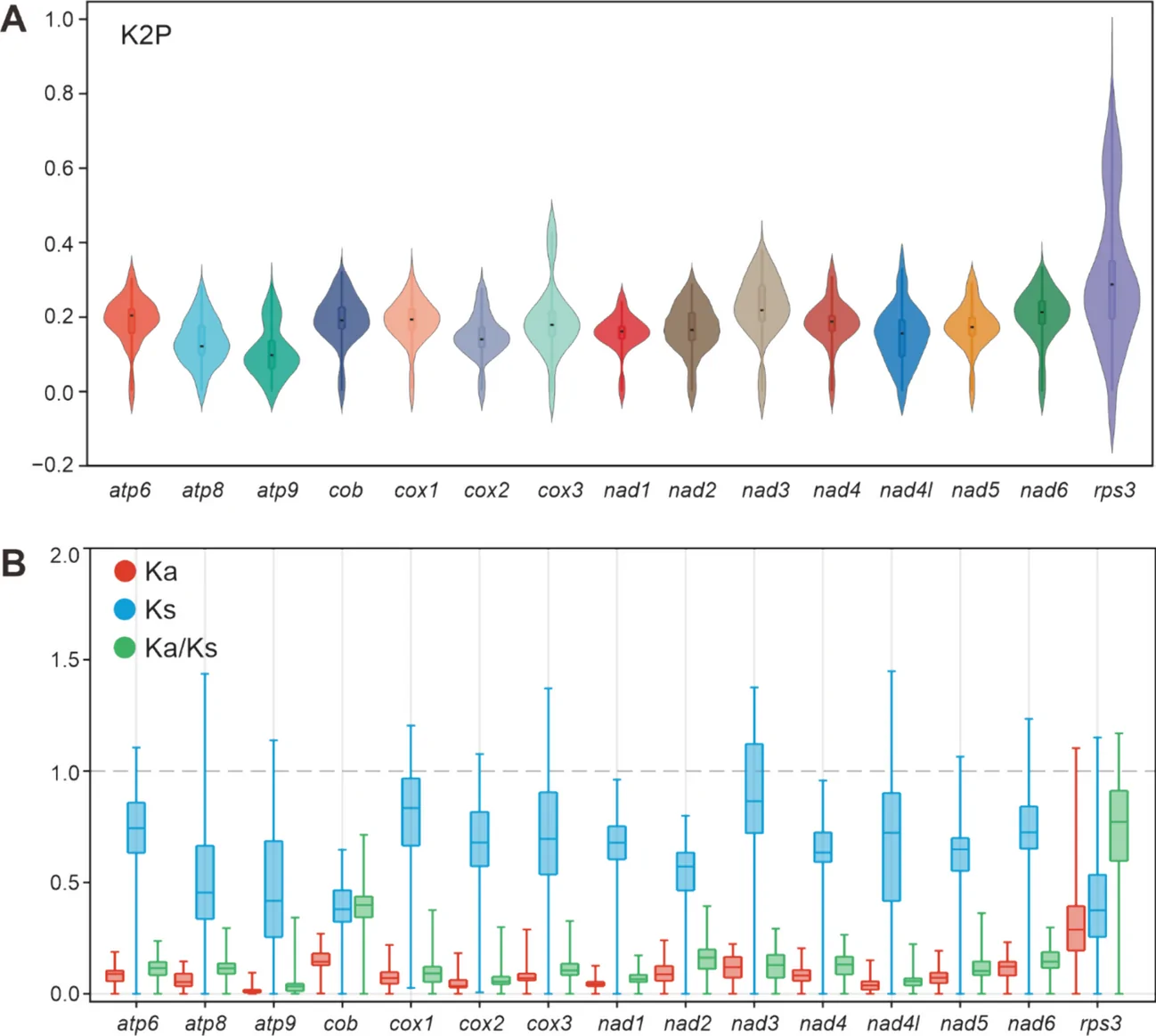
Genetic analysis of 14 conserved protein-coding genes and ribosomal protein (*rps3*) in Sanghuang-related species. **A** K2P, Genetic distance calculated with Kimura-2-parameter) and **B** Ka, the mean number of non-synonymous substitutions per non-synonymous site; Ks, the mean number of synonymous substitutions per synonymous site; Ka/Ks, which is used to estimate the balance between neutral mutations, purifying selection and beneficial mutations acting on a set of homologous protein-coding genes

Among the 15 detected core PCGs, *rps3* has the largest average K2P genetic distance in 16 Sanghuang-related mushrooms, followed by *nad3* and *nad6*, indicating that these genes, especially *rps3*, had undergone significant divergence during evolution. The *atp9*, conversely, exhibits the smallest K2P genetic distance (Fig. 4A, Table S8), which suggests that this gene was highly conserved, likely due to its role in energy supply in mitochondria. Further evolutionary pressure selection analysis showed that among the 15 core PCGs, the *rps3* gene had the highest Ka, while *atp9* had the lowest Ka value, similar to K2P. The Ks of *nad3* genes was the highest, while that of the *rps3* was the lowest. In general, the more diverse the species involved in the Ka/Ks calculation, the more likely the *rps3* ratio will be greater than 1. Although Sanghuang-related fungi were mainly distributed in four genera, the ratio of *rps3* was only 0.78 (Fig. 4B). This observation indicated that the *rps3* genes were not exposed under positive selection pressure among these similarly shaped Sanghuang-related fungi, and further implied that they were still under phylogenetic differentiation, with partial monophyly.

### Codon usage analysis

**Fig. 5:**
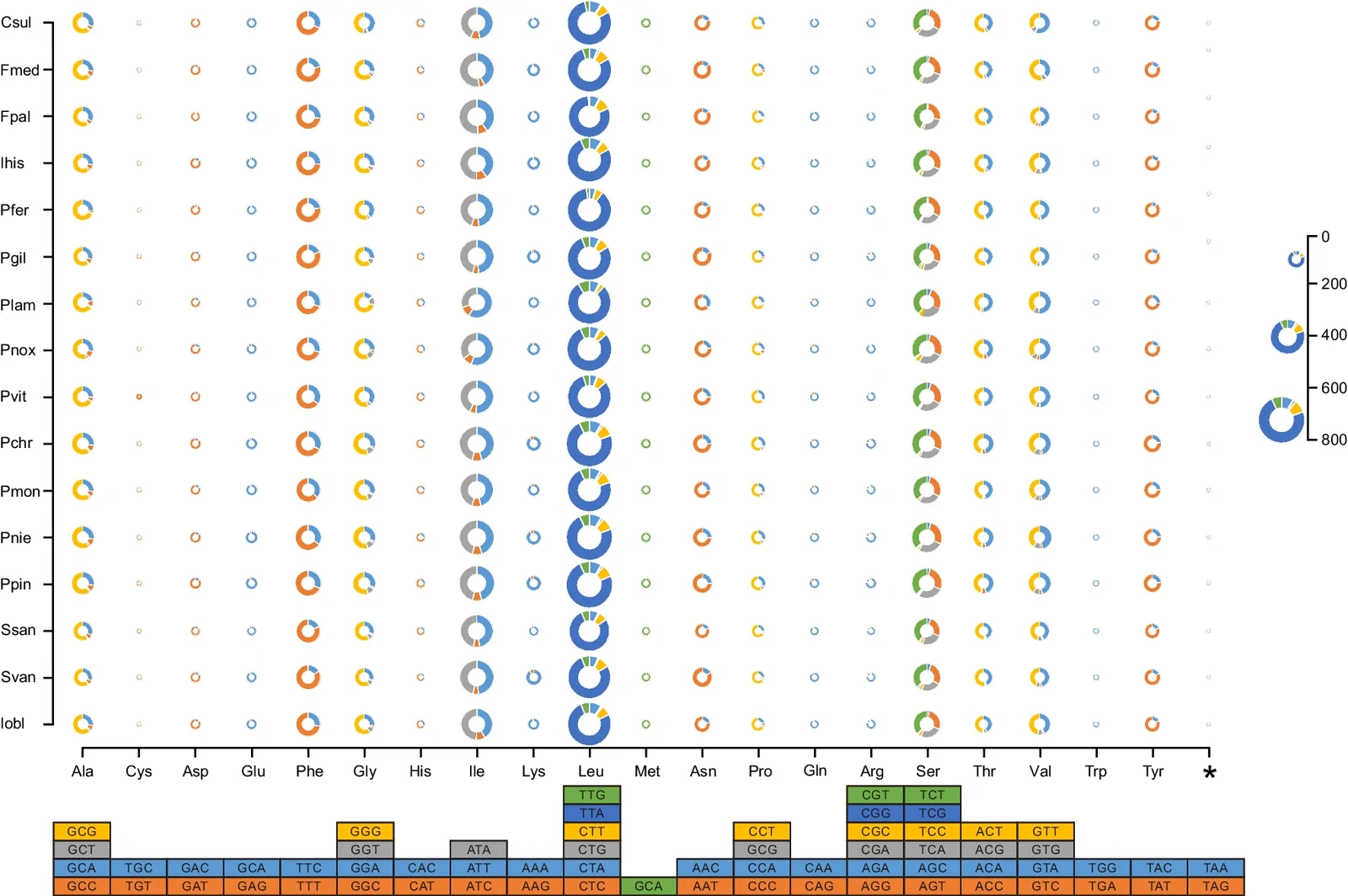
Codon usage of 14 conserved protein-coding genes and ribosomal protein (*rps3*) of the 16 species. The size of the ring indicates the ability to translation and the ring stacking parts represent the usage of different codons while “*” means stop codons

In order to examine the codon usage patterns of various mitogenomes of Sanghuang-related mushrooms, we compared a total of 16 mitogenomes including six annotated mitogenomes by our team. The *nad* genes, particularly *nad2* and *nad3*, exhibited a higher frequency of TTG as the start codon than other genes, while the distribution of stop codons appeared to be more even in the *nad* and *cox* genes. Among the core protein-coding genes of the 16 mitogenomes analyzed, TAA was the most frequently utilized stop codon, followed by TAG (Fig. 5, Table S9).

Further analysis revealed considerable variation in the usage of start and stop codons among Sanghuang-related species, even those with close phylogenetic relationships. For instance, the *nad3* and *cox2* genes of *P. pini* used TTG as their start codon, whereas other *Phellinus* species utilized ATG. Furthermore, the *nad6* of *P. viticol*a used TAG as a stop codon, while other *Porodaedalea* species utilized TAA (Fig. 5, Table S9). Therefore, it is necessary to verify the inferences of phylogenetic relationships based on codon usage.

Moreover, a detailed analysis revealed that UUA (leucine) was the most frequently used codons in Sanghuang-related mitogenomes, closely followed by UUU (phenylalanine) and AUU (isoleucine). Among these 16 mitogenomes, the most frequently translated amino acid was leucine (719 on average), while cysteine was the least translated (33 on average) (Fig. 5, Table S10). In summary, the codon usage bias of mitogenomes among sanghuang-like species was similar, which is consistent with the general rule that mitogenomes of the same order usually exhibit the same codon usage bias (Novoa and Ribas de Pouplana 2012). However, some exceptions may be due to the diversity of uORFs in mitogenomes.

### Assessment of uORF coding potential based on molecular signatures

**Fig. 6:**
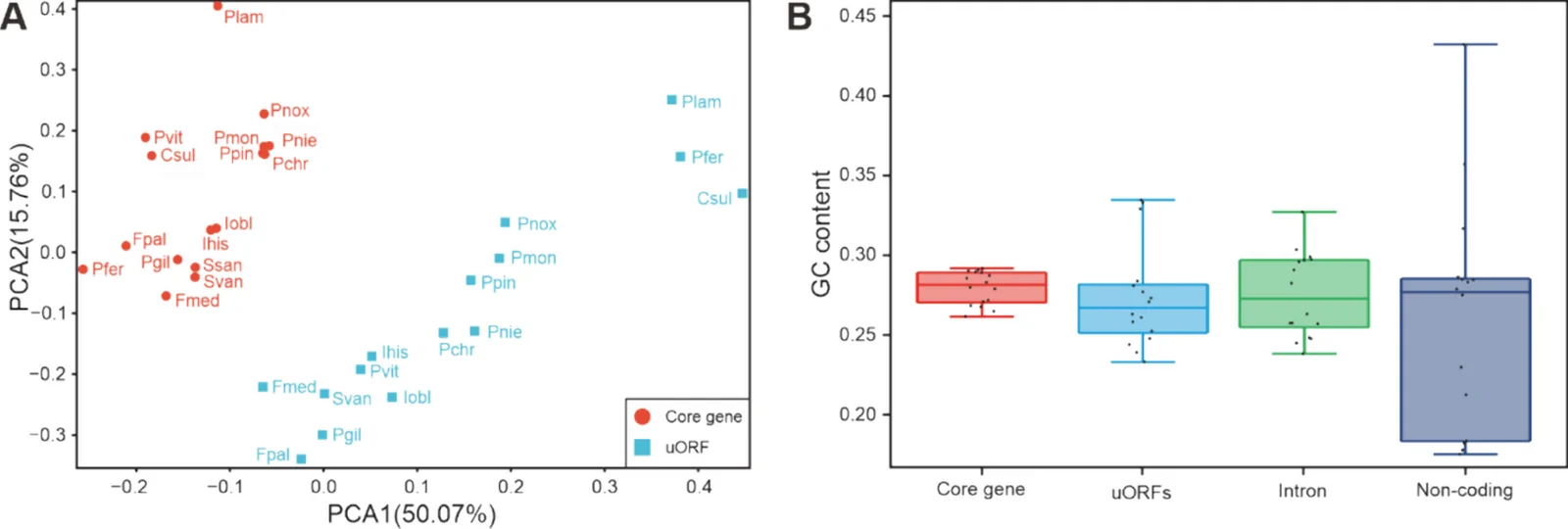
**A** Clustering analysis of Relative synonymous codon usage (RSCU) in core genes and uORFs of Sanghuang-related species. **B** GC content of four mitochondrial core genes, uORFs, introns and non-coding regions

The PCA revealed that the RSCU values of PCGs differed significantly from those of uORFs, with PCGs and uORFs clearly separated into two distinct clusters (Fig. 6A, Table S7&S10). This observation implies that PCGs are relatively conserved, while uORFs are more dynamic, and that the two evolutionary processes are independent of each other. This finding shed light on the convergent evolution of Sanghuang-related fungi in similar habitats.

The process of asymmetric replication in mitochondrial genomes results in differing mutation rates in the forward and reverse strands, thus affecting codon usage. AT-rich mitochondrial genomes have been shown to preferentially use AT-rich codons for gene encoding (Singer and Hickey 2000), and therefore, GC content is a crucial factor that influences codon usage. To further investigate the effect of GC content on codon usage, we performed a comparative analysis of GC contents in PCGs, uORFs, and intron regions. The results indicated a significant difference between the GC contents of core PCG regions and uORFs (*P* < 0.05) (Fig. 6B, Table S7), which suggests the potential use of GC content as a protein-coding marker.

### Intron dynamics of cox1 genes and gene rearrangements

**Fig. 7:**
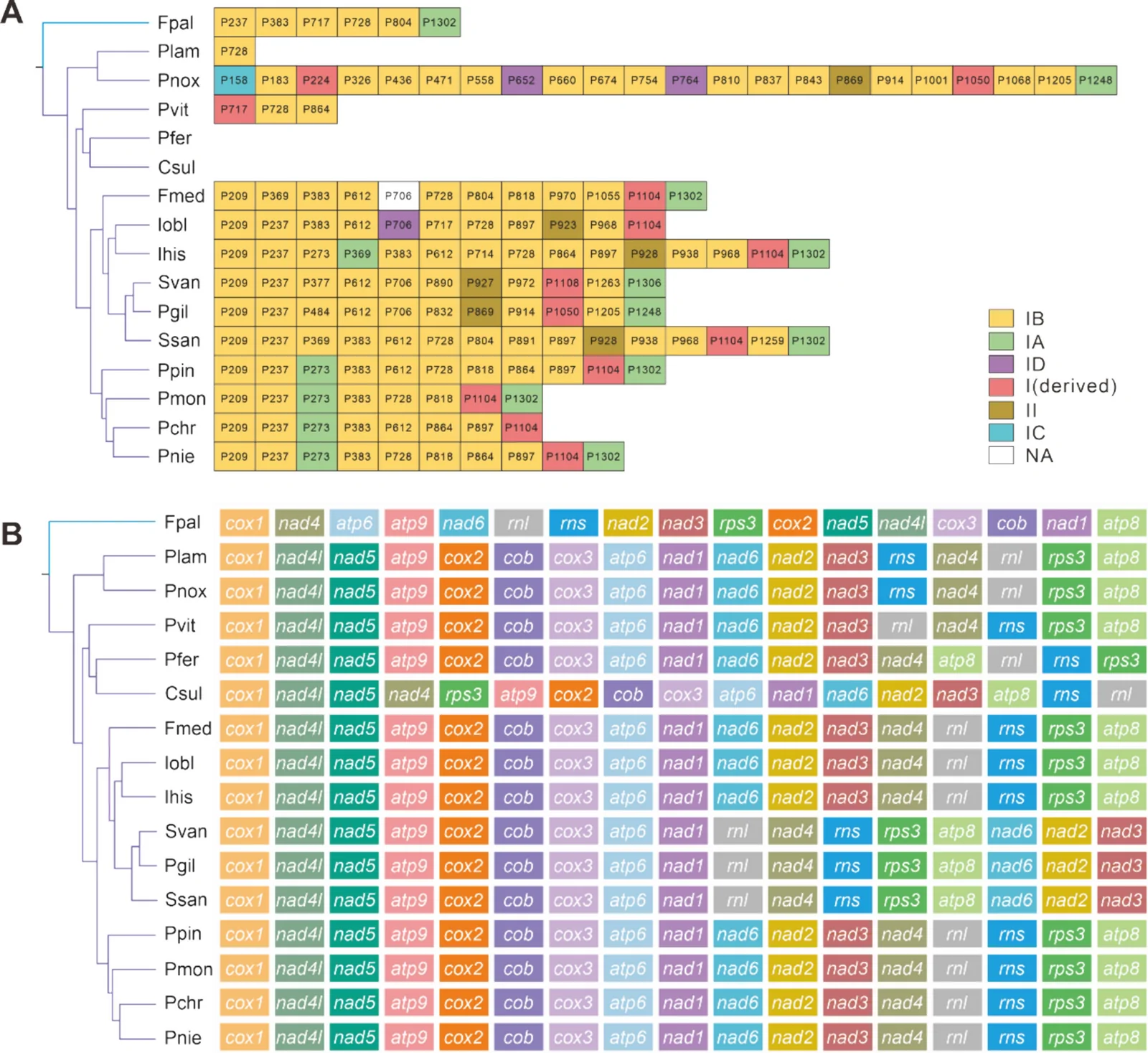
**A** Insertion position class (Pcl) and types of introns in *cox1* genes of Sanghuang-related species. The phylogenetic tree left is conducted based on AA dataset. **B** Mitochondrial gene arrangement of 15 core genes and ribosomal subunit genes (*rnl* and *rns*). Genes are represented with different color blocks. All genes are shown in order of occurrence in the mitochondrial genome, starting from *cox1*

The variation of introns in the *cox1* gene could significantly impact the length and structure of mitochondria. In total, 144 introns were found in *cox1* genes of 16 Sanghuang-related mushrooms. Position classes (Pcls) are used to define the same location of coding region in the *cox1* gene, and are often used to characterize the position information of introns contained in *cox1* gene (Ferandon et al. 2010). Pcls is evenly distributed on *cox1* gene of all species, with the exception of *P. ferrugineofuscus* and *C. sulphurascens*. The observed differences in intron class and number across the 16 species may indicate the occurrence of intron acquisition or loss. Pcls that occurred in no less than 20% of 16 species were designated as common introns, while others were defined as rare introns. All the introns detected in the *cox1* genes are classified into 54 Pcls, including 23 common Pcls and 31 rare Pcls. P209 and P728 are the most common Pcls appearing in 10 of the 16 species. P237 and P393 are found in 9 of the 16 mitogenomes as the second most common Pcls (Fig. 7A). Each kind of rare Pcls is only detected in a single species among the 16 species. Moreover, the types of introns in certain Pcls are also conserved among 16 *cox1* genes. For example, introns in P273 and P1302 are mostly type IA, introns in P1104 are all type I (derived). Interestingly, we further find that the intron in the latter part of PCGs is obviously conservative than that in the front part, no matter Pcls or intron types (Fig. 7A). *Sanghuangporus vaninii* and *P. gilvus* share more similar Pcls, intron types and even numbers of introns than *S. sanghuang*, implying their close relationship.

Large-scale gene rearrangements occurring at the family level and, even at the genus level, are common evolutionary events (Sankoff et al. 1992). Besides the differences in the content of mitogenomic components, the order of genes, including 15 core PCGs and two rRNA genes, varied considerably in all mitogenomes examined. Large-scale gene rearrangements, such as positional exchange and migration of genes, were observed in mitogenomes across *Sanghuangporus* and Sanghuang-like genera (Fig. 7B). It was also observed that a few gene rearrangements occurred in mitogenomes from *Phellinus* genera.

Gene rearrangements were observed in *Fomitopsis palustris* acting as an outgroup species, including gene inversion, insertion, and transfer events, which suggested that large-scale gene rearrangements occurred during the evolution. Notably, *nad4* and *rps3* were detected gene insertion between *nad5* and *atp9* in *C. sulphurascens* (Fig. 7B).

On the other hand, the certain gene order maintained in *S. sanghuang*, *S. vaninii*, and *P. gilvus*, which were arranged in the following orders: *cox1*, *nad4l*, *nad5*, *atp9*, *cox2*, *cob*, *cox3*, *atp6*, *nad1*, *rnl*, *nad4*, *rns*, *rps3*, *atp8*, *nad6*, *nad2*, and *nad3*, differing in those among *Porodaedalea*, *Inonotus*, and *Phellinus* species, seeming the clustering feature of *Sanghuangporus*.

### Sequence synteny analysis

**Fig. 8:**
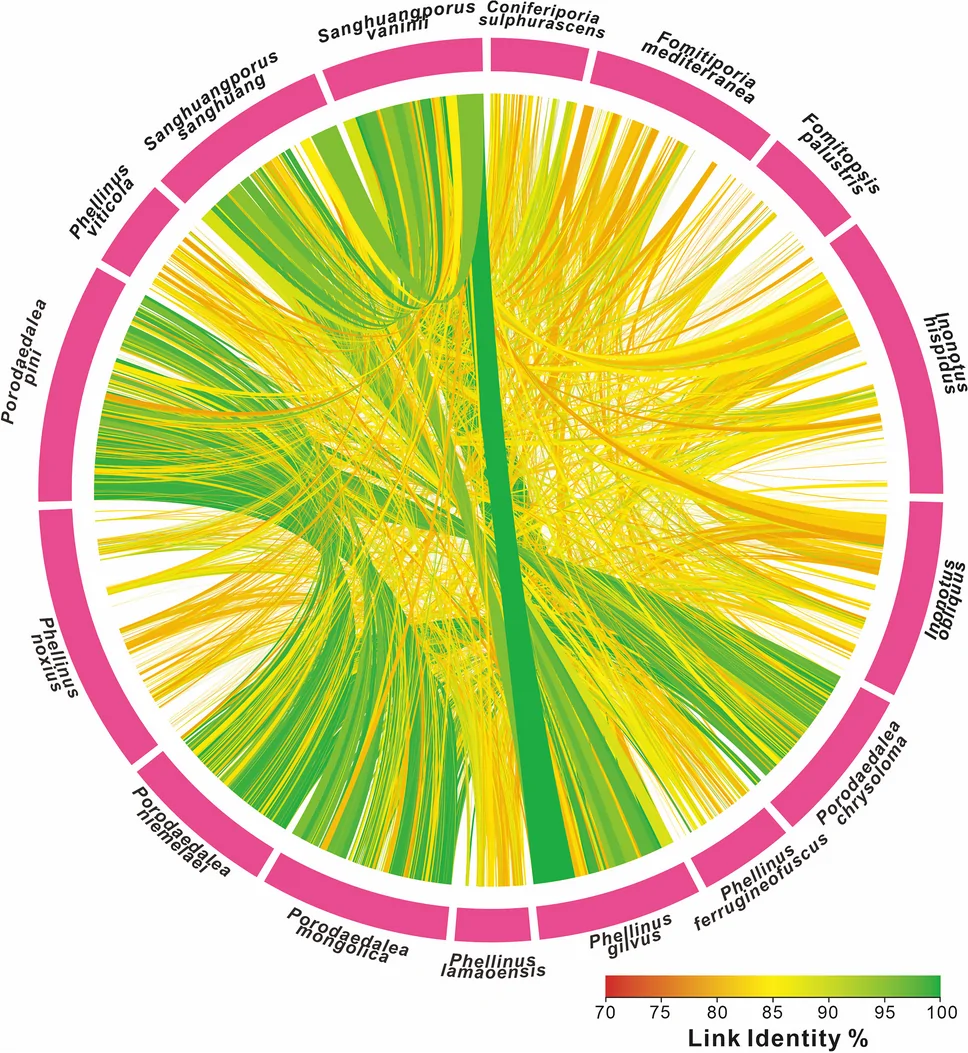
Mitogenome synteny among the 16 species links that identity > 70% was shown

To explore gene rearrangements in terms of the entire mitogenome, a synteny analysis based on 16 mitogenomes was performed. The results showed that scattered collinear sequences exist between most sanghuang-like species and large regions of identical sequence existed between the mitogenomes of *S. vaninii* and *P. gilvus*, and that multiple regions of high sequence identity also existed between them and *S. sanghuang*, respectively (Fig. 8). Additionally, the four mitogenomes of the distinctive genus from other species, *P. pini*, *P. niemelaei*, *P. mongolica*, and *P. chrysoloma*, exhibited multiple regions of high sequence identity with each other (Fig. 8). This situation, which occurs only among species within a genus, occurs among *Sanghuangporus* and *P. gilvus* S12, which indicates *S. vaninii* and *P. gilvus*, the two groups of Sanghuang-related fungi that exist in highly congruent regions may share closer relatives in terms of intragroup evolution. It also implies that *P. gilvus* S12 and *Phellinus* species are more distantly related compared to *Sanghuangporus* species.

### Phylogenetic analysis

**Fig. 9:**
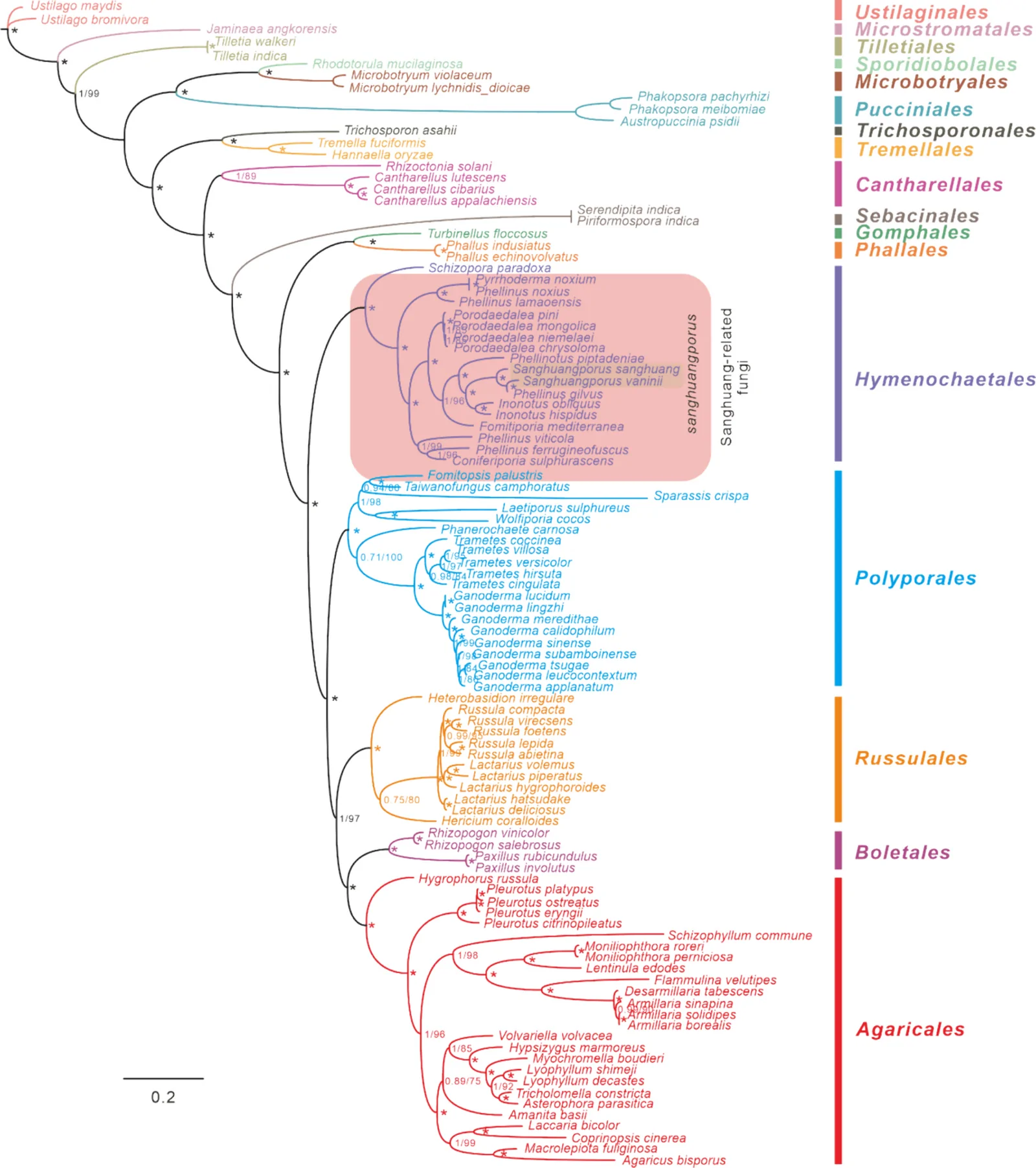
Phylogeny of 103 Basidiomycetes species based on amino acids of 14 conserved PCGs using BI and ML methods, generated by MrBayes v3.2.6 and IQ-TREE (v1.6.8). The numbers mean respectively Bayesian posterior probabilities (BPP) and bootstrap (BS) of the clade while asterisk means BPP value equals 1 and the BS value equals 100. The species and NCBI accession numbers for the mitogenomes used in the phylogenetic analysis are provided in Table S11

We performed an analysis of the amino acid set (3825 AA) of 103 Basidiomycete species, including representative species from Hymenochaetales, Polyporales, Russulales, and Agaricales. Two *Ustilago* species were used as out-groups (Fig. 9). The phylogenetic relationships of these species were consistently recovered as (Hymenochaetales + (Polyporales + (Russulales + (Boletales + Agaricales)))). All major clades within the trees were well supported. In particular, we discovered that Hymenochaetales are distantly related to other major basidiomycete species. This finding suggests that Hymenochaetales diverged early from these species, possibly representing an ancient lineage, while retaining a similar morphology to Polyporales. Interestingly, *F. palustris*, a member of Polyporales, was once considered to be a Sanghuang due to its morphological similarities.

**Fig. 10:**
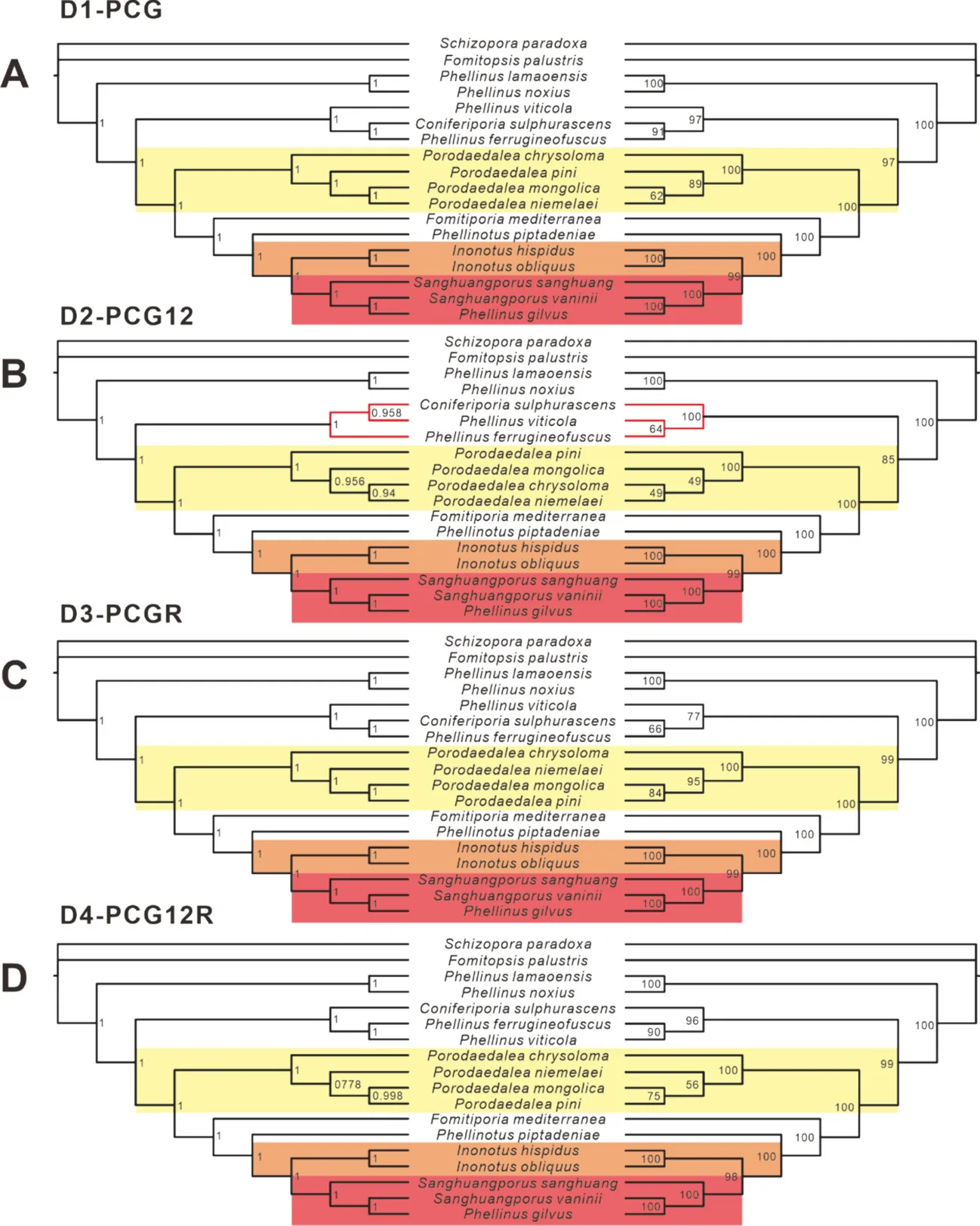
BI and ML trees conducted based on the four datasets: D1: PCG (**A**); D2: PCG12 (**B**); D3: PCGR (**C**); D4: PCG12R (**D**). The relatively conserved tribes are in different color blocks

The evolutionary rate of fungi is known to be faster than that of insects, with significant differences observed in PCGs and rRNA sequences across different families. In this study, we conducted a phylogenetic analysis using the PCG (12,936 bp), PCG12 (8624 bp), PCGR (16,532 bp), and PCG12R (12220 bp) datasets, respectively, from 16 Sanghuang-related species, instead of the amino acids of conserved genes in the 103 Basidiomycete mitogenomes, and taking *Fomitopsis palustris* as an out-group (Fig. 10, Table S12). Phylogenetic trees were conducted after the datasets having undergone heterogeneity analysis and base substitution saturation testing (Fig. S8-S9, Table S13). Our analysis revealed that the clades *Sanghuangporus* and *Inonotus* exhibited identical phylogenetic topologies ((*I. hispidus* + *I. obliquus*) + (*S. sanghuang* + (*S. vaninii* + *P. gilvus*))) across all four datasets, with strong support from high BPP (1) and BS (100) values (Fig. 10). However, for the *Porodaedalea* species, the PCGR and PCG12R datasets show the same taxonomic units (Fig. 10C, D), whereas the PCG (Fig. 10A) and PCG12 (Fig. 10B) datasets do not. Their lower BS values further indicate that the four species are indistinguishable. Our findings also revealed that *Phellinus* species are distributed broadly as a paraphyletic group in phylogenetic trees of the four datasets and AA dataset (Fig. 10). This situation may be due to the lax identification and naming of species with very similar morphology in previous research. In addition, we observed variations between *Coniferiporia sulphurascens* and *P. viticola*, with the PCG (Fig. 10A) and PCGR (Fig. 10C) datasets providing one topology and the PCG12 (Fig. 10B) and PCG12R (Fig. 10D) datasets providing another. Further differences were noted among the three species, *C. sulphurascens*, *P. viticola*, and *P. ferrugineofuscus.* The topologies of these three species existed in four BI trees and four ML trees existed in three cases. Two BI trees and two ML trees supported (*P. viticola* + (*C. sulphurascens* + *P. ferrugineofuscus*)) (Fig. 10), one BI tree and two ML trees supported (*C. sulphurascens* + (*P. viticola* + P. *ferrugineofuscus*)), and only one BI tree supported (*P. ferrugineofuscus* + (*P. viticola* + *C. sulphurascens*)). Of these, the first case had the highest number of trees and the highest node support.

*Sanghuangporus* subclade steadily maintain their monophyly from *Inonotus* and *Phellinus*, in agreement with Dai’s research (Shenghua et al. 2012). Compared to *S. sanghuang*, *S. vaninii* shows a closer relationship to *P. gilvus*, acting as a pair of sister species, making it worthwhile to discuss whether *P. gilvus* should be classified into *Phellinus*. The results indicated that the combined mitochondrial gene datasets were suitable as reliable molecular markers for the analysis of phylogenetic relationships among Basidiomycete species.

## Discussion

### Morphological confusion and misuse of Sanghuang-related fungi

Sanghuang, a medicinal mushroom renowned for its remarkable healing properties, has been widely referenced in various medical books and has been revered by ancient civilizations. Its use as a medicinal substance has a long history in countries such as Japan and Korea. However, traditional medical books have only provided simple descriptions of the morphology, medicinal properties, and habitat of Sanghuang, leading to the proliferation of numerous homonyms and synonyms. As a result, mushrooms such as *I. hispidus* (Bao et al. 2013; Hai-ying et al. 2017; Wang et al. 2022; Zhang et al. 2022), and *Phellinus linteus* (Chen et al. 2016; Hsieh et al. 2013) have been erroneously identified as Sanghuang, leading to much confusion and misinformation. The similarity in appearance, habits, and pharmacological effects of these mushrooms and the sanghuang has made it difficult to distinguish between them, even among experts. The discrepancies between the Sanghuang recorded in the pharmacopeia and those used in folk medicine have exacerbated this problem, as their very similar characteristics have left many people struggling for centuries to make the correct identification. Despite the recent establishment of the genus *Sanghuangporus* based on ITS, morphological characteristics and mycelial structure, distinguishing between *Sanghuangporus* and Sanghuang-like fungi remains a challenge due to their overlapping characteristics.

To address these concerns, we present a novel approach utilizing genetic molecular traits to differentiate between Sanghuangporus and Sanghuang-like mushrooms. We believe that mitogenomic analysis is a more reliable and abundant source of molecular information than ITS and can provide detailed insights into their phylogenetic relationships. The use of the mitogenome as a tool for studying phylogenetic relationships is particularly ideal due to its small length, genes conservatism, and genetic monolepsis, which enhances the accuracy and reliability of our findings.

### Comparative mitogenomic analysis of Sanghuang-related fungi

In the present study, we conducted a comparative mitogenomic analysis of 16 Sanghuang-related species and found that the molecular information among these species exhibited significant variability. The mitogenome size of Sanghuang-related fungi ranging from 45,604 bp to 170,878 bp, indicating that their genetic composition differed substantially. In particular, we observed a higher proportion of *nad4* in *Porodaedalea* fungi (Fig. 2A), suggesting that they have undergone distinct phylogenetic differentiation. Furthermore, the low value of *rps3* in the 16 species (Fig. 4B) suggested a close genetic affinity between these fungi. Notably, we observed significant differences in GC content (Fig. 3B) and AT skew (Fig. 3D) among the 16 species, which were relatively dispersed throughout their mitogenomes. Additionally, GC density displayed a marked difference between ORFs and uORFs (Fig. 6B), implying that mitochondrial non-core genes are more active than core genes. Taken together, our findings reveal that the non-core regions of Sanghuang-related fungi mitogenomes exhibit greater diversity and activity than their core genes.

The present study provides compelling evidence for convergence and congruence among three Sanghuang-related fungi: *S. sanghuang*, *S. vaninii* and *P. gilvus*. First, their mitogenome sizes (Fig. 2), as well as the sizes of their 14 PCGs and *rps3* (Fig. 3A) were found to be similar. In addition, PCA of the RSCU of uORFs revealed a significant convergence of the three species compared to other fungi (Fig. 6A). Interestingly, intron analysis of *cox1* revealed identical intron profiles for *S. vaninii* and *P. gilvus*, which were very similar to those of *S. sanghuang* (Fig. 7A). Gene rearrangement analysis showed that the gene order of the three species was the same, distinguishing them from other fungi (Fig. 7B). Finally, synteny analysis of mitogenomic DNA revealed large regions of high sequence similarity between the three species (Fig. 8). These observations suggest a close evolutionary relationship between *S. sanghuang*, *S. vaninii*, and *P. gilvus* and provide new insights into the mechanisms underlying their convergent evolution.

Our finding sheds light on the diversity and complexity of mitochondrial genomes in Sanghuang-related fungi. Although local convergence and uniformity were observed in *S. sanghuang*, *S. vaninii*, and *P. gilvus*, the molecular information among Sanghuang-related species was found to be inconsistent in several aspects. These results provide new insights into the molecular basis for distinguishing *Sanghuangporus* species from Sanghuang-related fungi. However, the analysis of codon usage preferences and expression requirements revealed a conservative pattern among the Sanghuang-related species (Fig. 5). Interestingly, leucine, serine, and arginine had the highest number of codons with six, while the corresponding tRNAs (*trnL*(tag/taa), *trnS* (gct/tga), and *trnR* (tct/tcg)) all had two. The copy number of *trnM* (for Methionine) in all six species is 3, and the anticodon of all three copies of *trnM* is CAU (Tables S2). This observation suggests a coordinated protein translation process in the mitochondria of mulberry-like fungi. Therefore, codon usage may not be the most appropriate marker to distinguish *Sanghuangporus* species from other Sanghuang-related fungi.

### Mitogenome-based phylogenetic analysis of Sanghuang-related fungi

The construction of a phylogenetic tree using amino acid sequences of 14 PCGs in the mitogenomes of 103 Basidiomycetes revealed that Sanghuang-related fungi are primarily grouped within the Hymenochaetales clade. Notably, *Sanghuangporus*, as a member of the Sanghuang-related group, forms an independent branch that serves as a sister group to *Inonotus* species (Fig. 9). In contrast, members of the genus *Phellinus* are found in different subclades rather than forming a monophyletic group (Fig. 9). This observation suggests that the current classification of the genus *Phellinus* may not be based on molecular characters but rather on morphological similarities. These results shed light on the evolutionary relationships among Basidiomycetes, in particular the need to re-evaluate the classification of the genus *Phellinus* based on molecular evidence.

Due to codon degeneracy, amino acid-based phylogenetic trees may not capture differences at the codon level. Therefore, to gain a more comprehensive understanding of the phylogenetic topology within Sanghuang-related fungi, we constructed four additional phylogenetic trees using corresponding datasets. These analyses revealed that the three main genera of Sanghuang-related fungi, *Porodaedalea*, *Inonotus*, and *Sanghuangporus*, shared overall fixed topologies with high support (Fig. 10). However, the internal topology of *Porodaedalea* exhibited considerable variation across the four phylogenetic trees (Fig. 10). Moreover, the phylogenetic trees constructed based on the five datasets revealed that *Inonotus* and *Sanghuangporus* form sister groups, indicating that *Inonotus* has the closest evolutionary relationship with *Sanghuangporus* among Sanghuang-like fungi. These findings provide novel insights into the phylogenetic relationships among Sanghuang-related fungi and highlight the importance of considering codon-level variations when constructing phylogenetic trees.

Notably, we found that the three species, *S. sanghuang*, *S. vaninii*, and *P. gilvus*, were consistent not only in gene arrangement, intron dynamics, and synthesis analysis, but also in phylogenetic analysis based on various datasets. Furthermore, *P. gilvus* is more closely related to *S. vaninii* than to *S. sanghuang*. These results make it more reasonable to speculate that *P. gilvus* is a species of *Sanghuangporus*. In fact, the *P. gilvus* S12 used in this study is an ear-shaped yellow mushroom collected from a growing mulberry tree, and its main active ingredient, styrylpyrones, is also abundant in *S. vaninii* (Song et al. 2023) and *S. sanghuang* (Jin-Jin et al. 2021). Therefore, we speculate *P. gilvus* S12 may represent a new species of *Sanghuangporus* parasitic on mulberry trees. Indeed, the discoverers of strain S12 have also recently reclassified it and named it *S. vaninii* S12 (Huo et al. 2022; Shen et al. 2021). These results provide novel insights into the evolutionary relationships of closely related fungi and suggest new avenues for further exploration of the diversity of *Sanghuangporus* species.

In the present study, we assembled and annotated six mitogenomes of *I. hispidus*, *P. gilvus*, *P. niemelaei*, *P. ferrugineofuscus*, *P. chrysoloma*, and *P. viticola* from raw sequencing data. The comparative mitogenomic investigation of the 16 Sanghuang-related fungi revealed high similarity among Sanghuang-related species in terms of mitogenome characterization, sequence features, gene distance, Ka/Ks, and RSCU. Nonetheless, intron dynamics, gene rearrangement, and synteny analysis exposed unique features of *Sanghuangporus* fungi, which helped to distinguish *Sanghuangporus* from Sanghuang-like fungi. Phylogenetic analyses based on five datasets of 16 Sanghuang-related fungi demonstrated the overall evolutionary position of Sanghuang-related fungi among Basidiomycete fungi. Furthermore, the analyses revealed the monophyly of *Sanghuangporus*, *Inonotus*, and *Porodaedalea* among Sanghuang-related fungi, with *Sanghuangporus* and *Inonotus* as sister taxa on the most complex branch of the Sanghuang-related species. The study also provided new insights into the species grouping of *P. gilvus* S12. The presented mitochondrial-based molecular evidence provides novel means for distinguishing *Sanghuangporus* and serves as a critical reference for resolving nomenclatural confusion in Sanghuang-related species.

## Supplementary Information

Below is the link to the electronic supplementary material.

Supplementary file1 (PDF 4381 KB)

Supplementary file2 (XLSX 304 KB)

## Data Availability

The complete mitogenomes of *Inonotus hispidus*, *Phellinus gilvus*, *Phellinus viticola*, *Porodaedalea chrysoloma*, *Phellinus ferrugineofuscus*, and *Porodaedalea niemelaei*, were deposited in the GenBank database under the accession numbers ON969135, OP265749, OP141808, OP141806, OP141805, and OP141804, respectively. The wild-type strain of *Phellinus gilvus* and *Inonotus hispidus* has been deposited in China General Microbiological Culture (CGMCC) under the deposited number CGMCC 11403 and Agricultural Culture Collection of China (ACCC) under the deposited number ACCC 35518.
