# Supplementary material for: Distinguishing Sanghuangporus from sanghuang-related fungi: a comparative and phylogenetic analysis based on mitogenomes
Source: Appl Microbiol Biotechnol. 2024 Jul 22;108:423. doi: 10.1007/s00253-024-13207-1 (PMC11263249; doi:10.1007/s00253-024-13207-1)
Supplement: Supplementary file 1 [file 253_2024_13207_MOESM1_ESM.pdf]

Distinguishing *Sanghuangporus* from Sanghuang-related Fungi: A Comparative and Phylogenetic Analysis based on Mitogenomes

Xi-long Feng [a], Tian-chen Xie [a], Zhen-xin Wang [a], Chao Lin [a], Zhao-chen Li [a], Jinxi Huo [b], Yougui Li [b], Chengwei Liu [c], Jinming Gao [a], Jianzhao Qi [a,] *

[a] Shaanxi Key Laboratory of Natural Products & Chemical Biology, College of Chemistry & Pharmacy, Northwest A&F University, Yangling, Xianyang 712100, China

[b] Sericultural Research Institute, Zhejiang Academy of Agricultural Sciences, Hangzhou 310021, People's Republic of China

[c] Key Laboratory for Enzyme and Enzyme-like Material Engineering of Heilongjiang, College of Life Science, Northeast Forestry University, Harbin 150040, China

Corresponding author: Jianzhao Qi, qjz@nwafu.edu.cn, ORCID: 0000-0003-1418-873X

Content

Figure S1. Predicted secondary cloverleaf structures for tRNAs of *Inonotus hispidus*. 1

Figure S2. Predicted secondary cloverleaf structures for tRNAs of *Porodaedalea chrysoloma*. 2

Figure S3. Predicted secondary cloverleaf structures for tRNAs of *Phellinus ferrugineofuscus*......3

Figure S4. Predicted secondary cloverleaf structures for tRNAs of *Phellinus gilvus*.......................4

Figure S5. Predicted secondary cloverleaf structures for tRNAs of *Porodaedalea niemelaei*. 5

Figure S6. Predicted secondary cloverleaf structures for tRNAs of *Phellinus viticola*. 6

Figure S7. Mitochondrial relative composition of all Sanghuang-related fungi and circular maps of the other 10 Sanghuang-related fungi. 7

Figure S8. Heterogeneous sequence differentiation of mitochondrial genome based on the four data sets. 8

Figure S9. Base substitution saturation analysis based on the four datasets, conducted by DAMBE[3]. 9

Reference 10

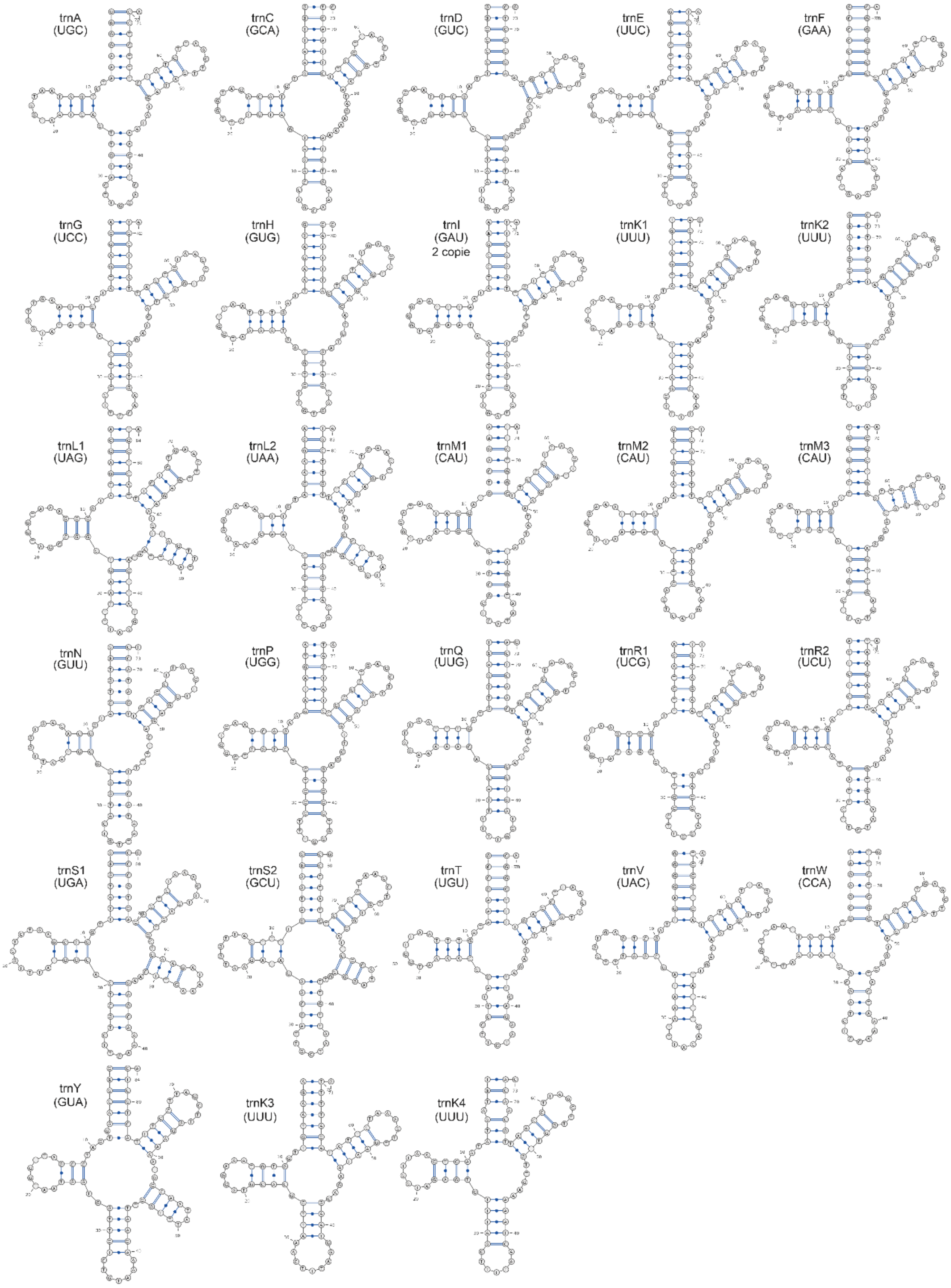

Figure S1. Predicted secondary cloverleaf structures for tRNAs of *Inonotus hispidus*.

(A) Isoleucine, (B) Glutamine, (C) Methionine, (D) Tryptophan, (E) Cysteine, (F) Tyrosine, (G, U) Leucine, (H) Lysine, (I) Aspartic, (J) Glycine, (K) Alanine, (L) Arginine, (M) Asparagine, (N, T) Serine, (O) Glutamic, (P) Phenylalanine, (Q) Histidine, (R) Threonine, (S) Proline, (V) Valine. The image was predicted by tRNAscan-SE 2.0.9(Chan and Lowe 2019) (http://lowelab.ucsc.edu/tRNAscan-SE), drawing with VARNA (http://varna.lri.fr/).

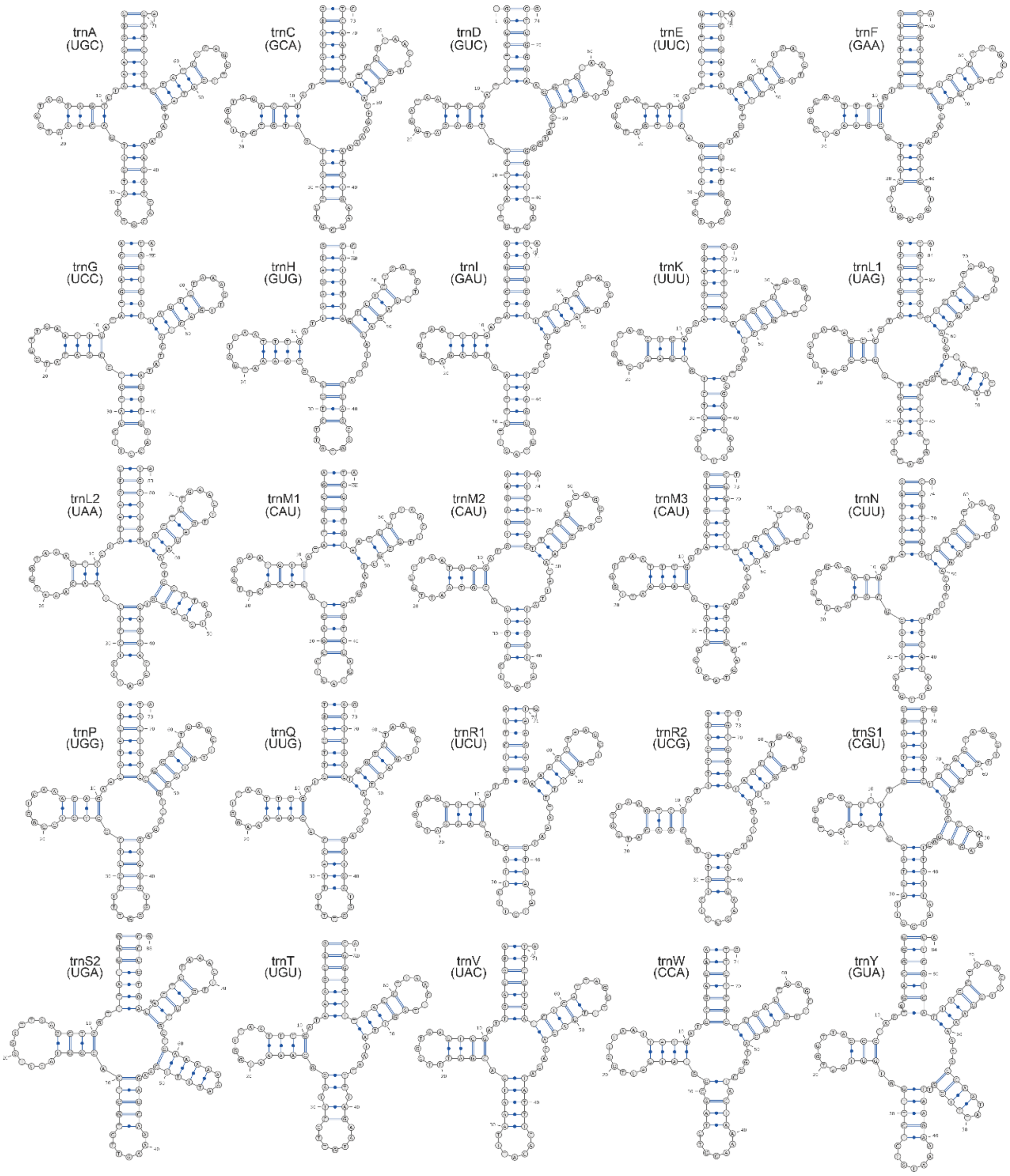

Figure S2. Predicted secondary cloverleaf structures for tRNAs of *Porodaedalea chrysoloma*.

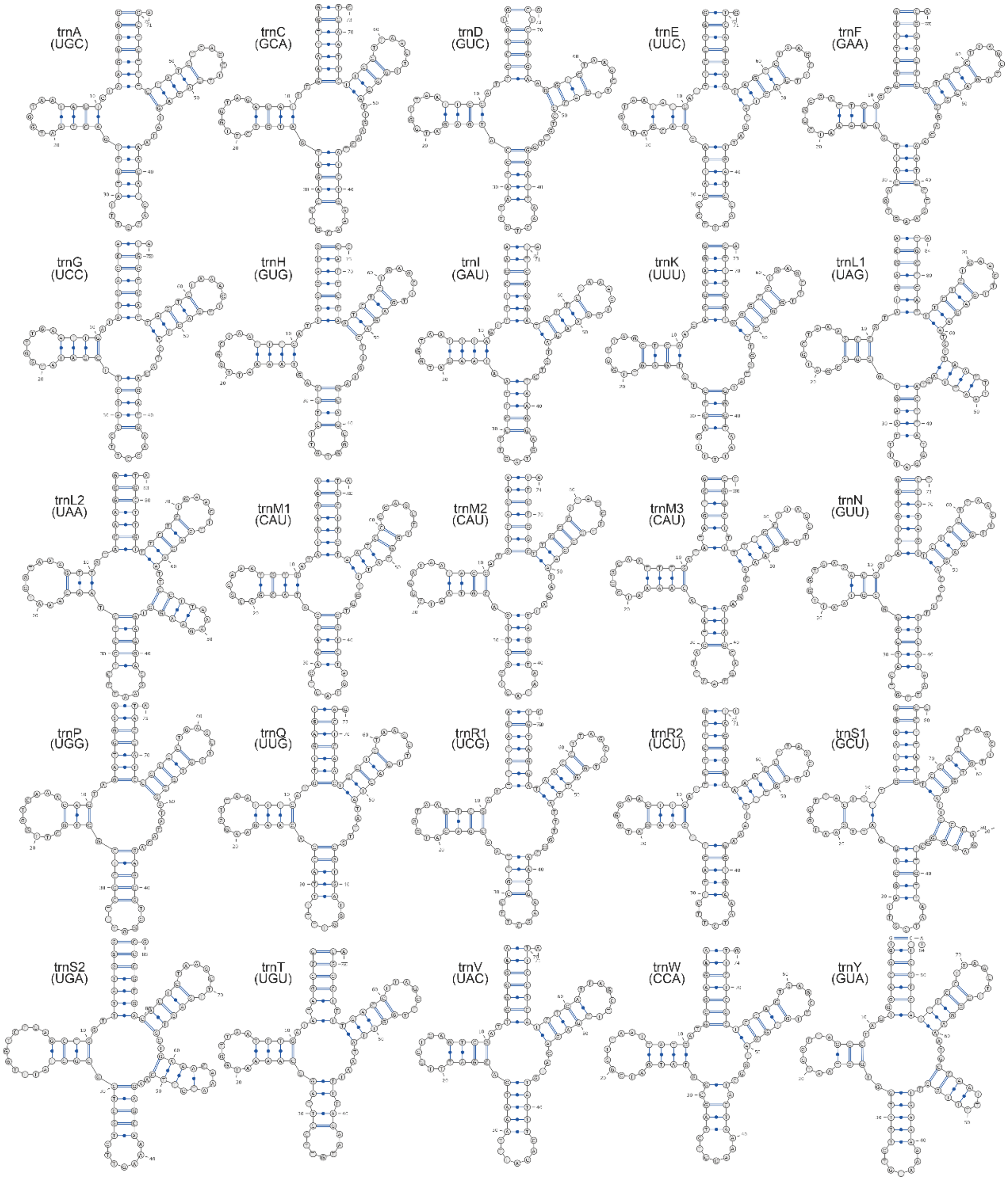

Figure S3. Predicted secondary cloverleaf structures for tRNAs of *Phellinus ferrugineofuscus*.

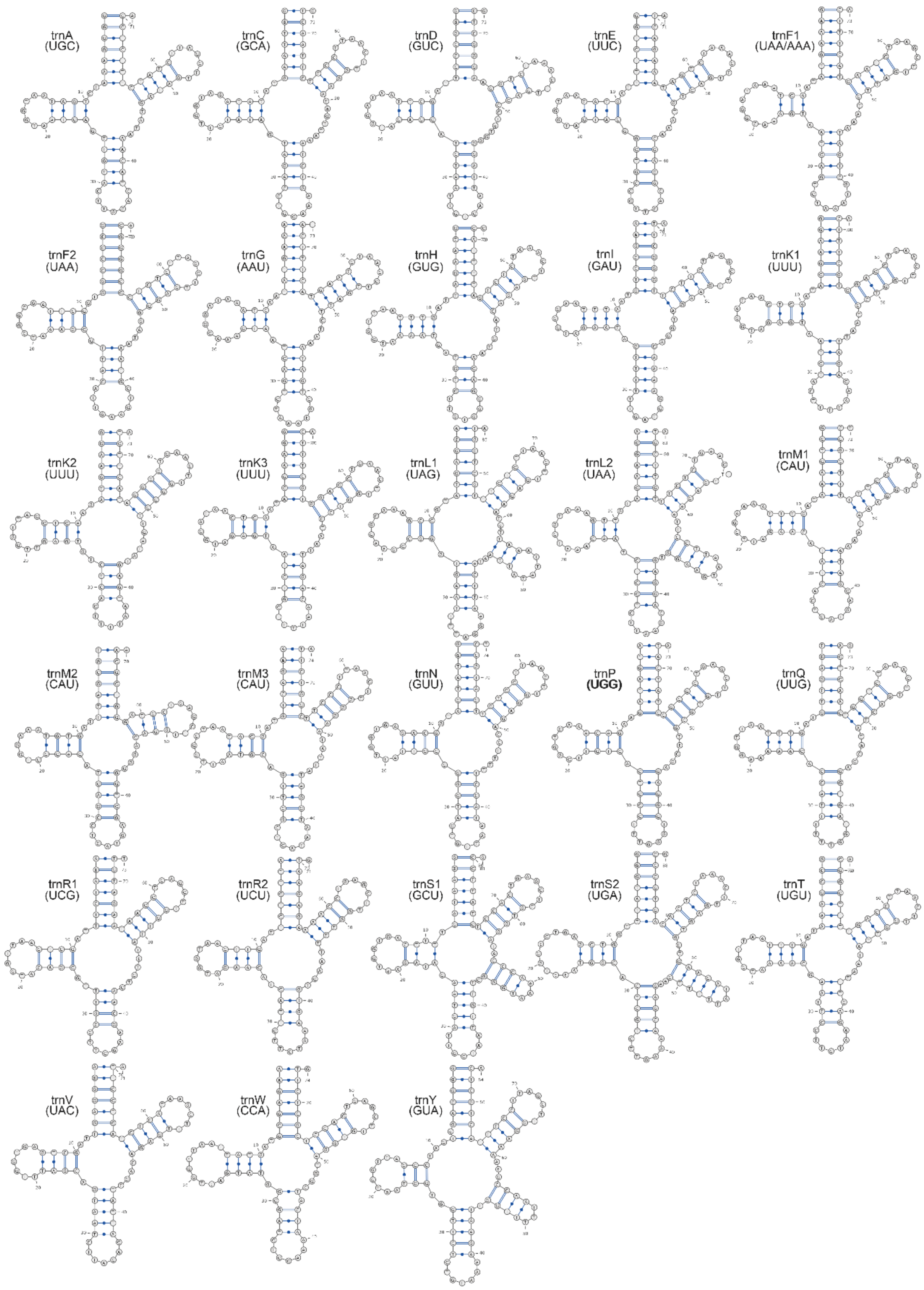

Figure S4. Predicted secondary cloverleaf structures for tRNAs of *Phellinus gilvus*.

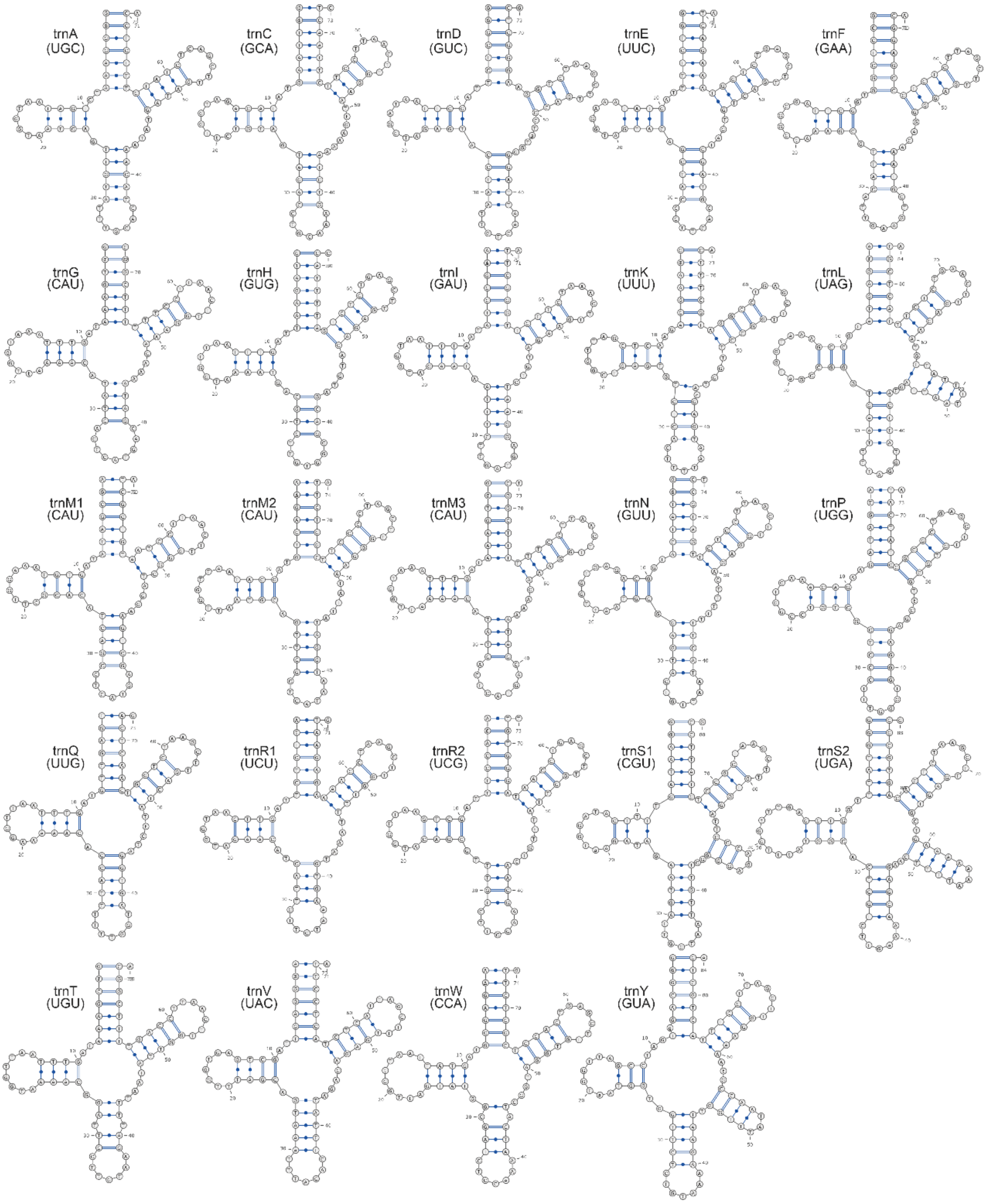

Figure S5. Predicted secondary cloverleaf structures for tRNAs of *Porodaedalea niemelaei.*

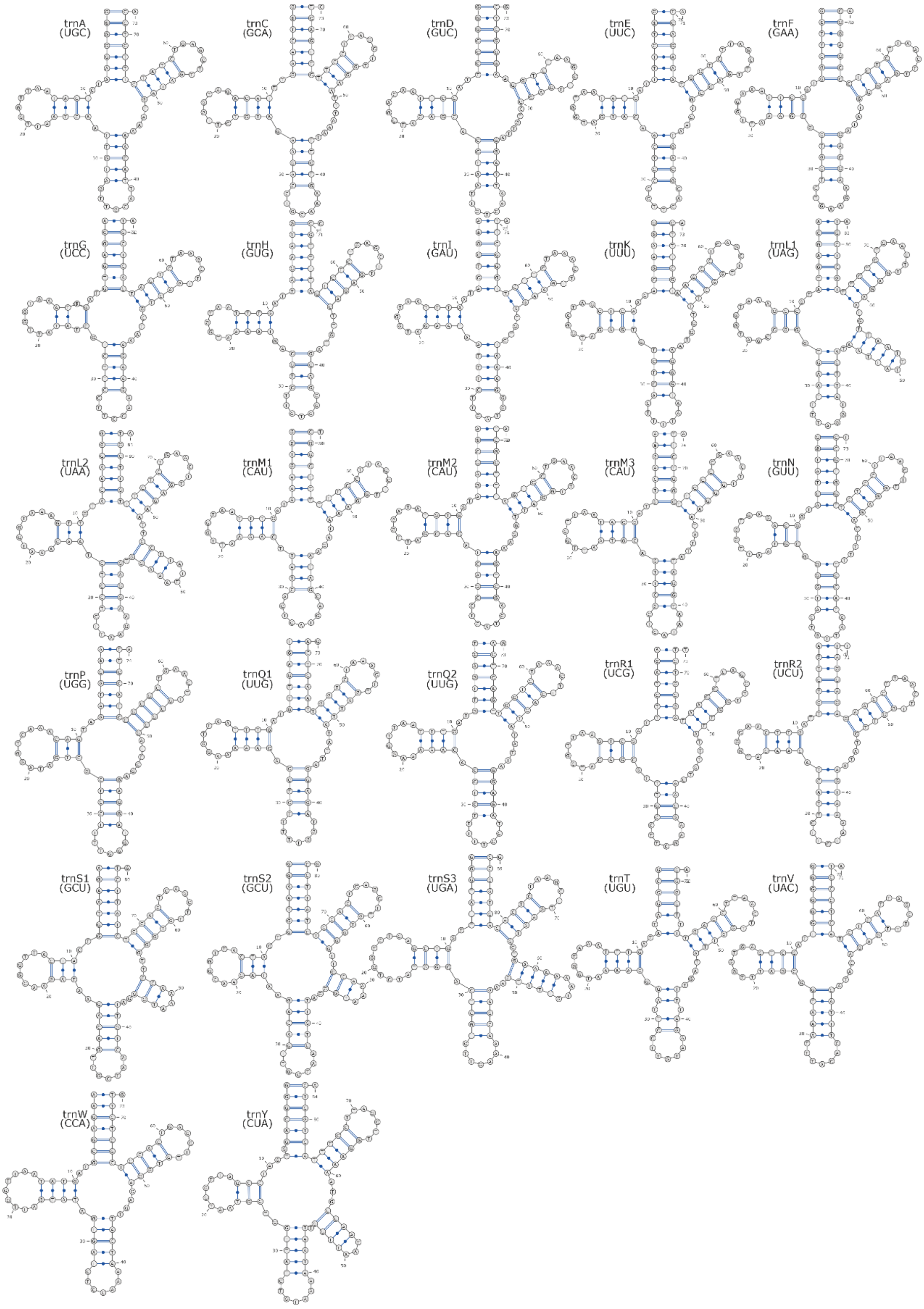

Figure S6. Predicted secondary cloverleaf structures for tRNAs of *Phellinus viticola*.

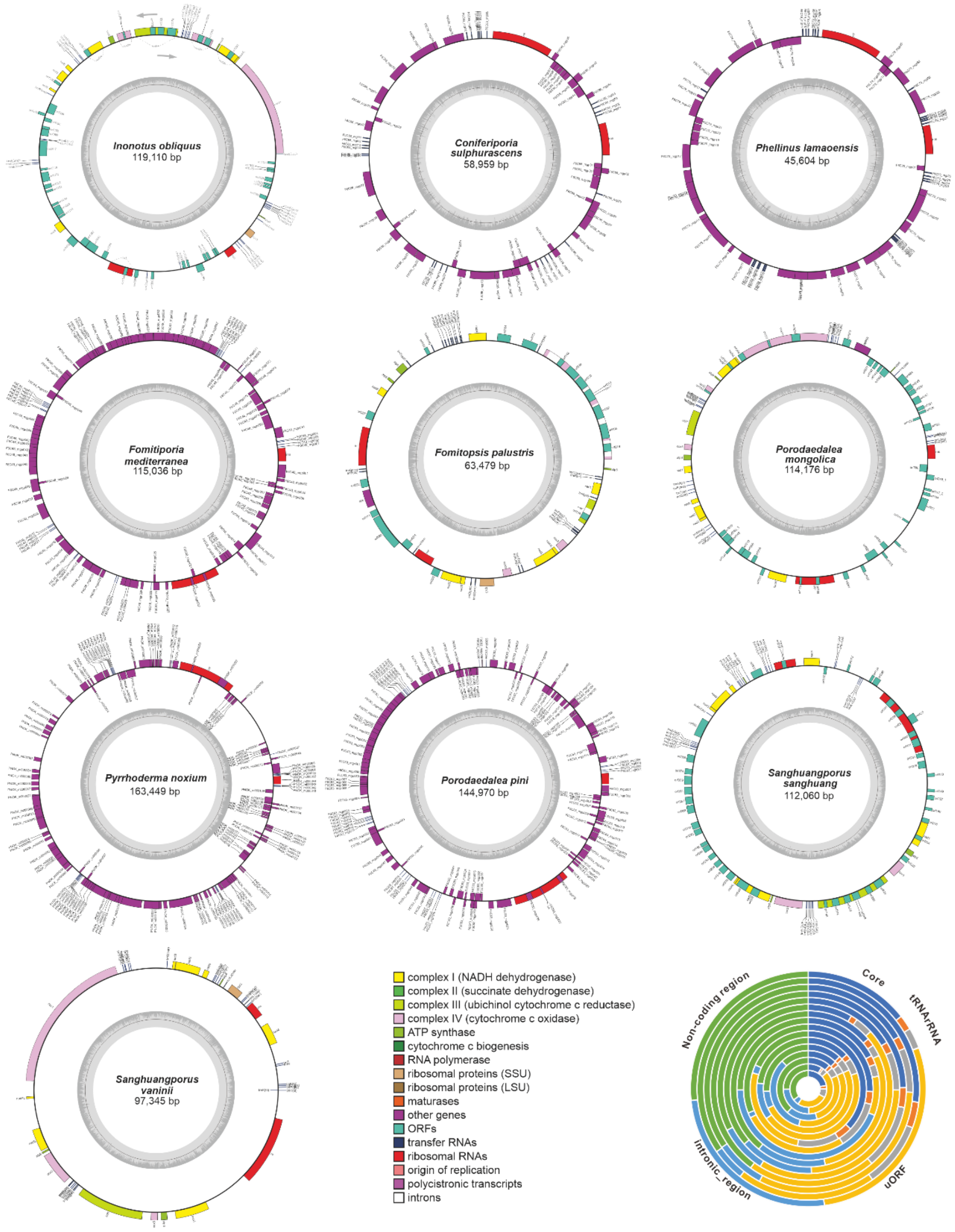

Figure S7. Mitochondrial relative composition of all Sanghuang-related fungi and circular maps of the other 10 Sanghuang-related fungi.

These Sanghuang-related fungi includes *Inonotus obliquus, Coniferiporia sulphurascens, Phellinus lamaoensis, Fomitiporis mediterranea, Fomitopsis palustris, Porodaedalea mongolica, Pyrrhoderma noxium, Porodaedalea pini, Sanghuangporus sanghuang and Sanghuangporus vaninii.*

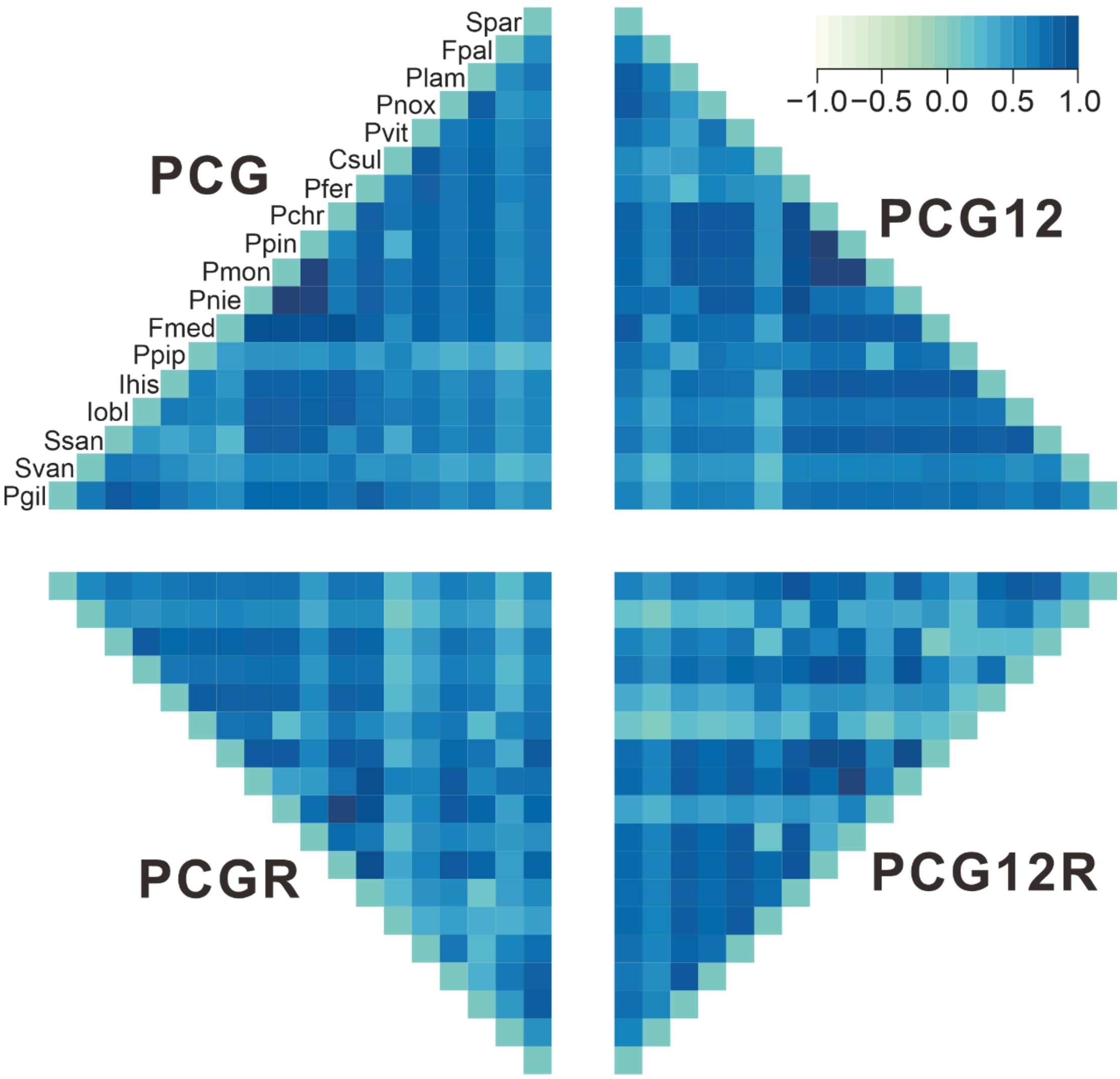

Figure S8. Heterogeneous sequence differentiation of mitochondrial genome based on the four data sets.

The average similarity score between the sequences is represented by a colored block, based on an AliGROOVE(Kück et al. 2014) score from -1 to +1.

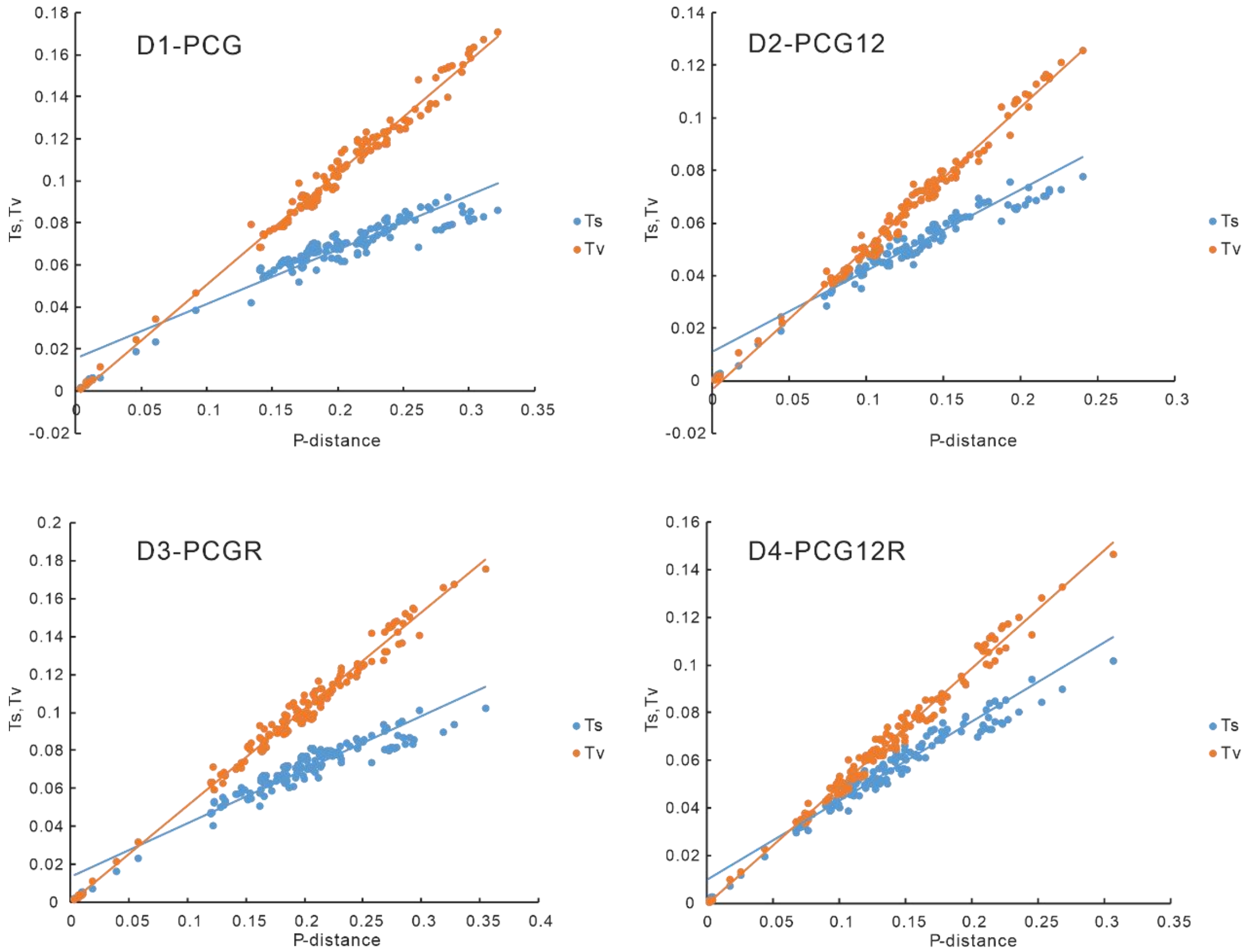

Figure S9. Base substitution saturation analysis based on the four datasets, conducted by DAMBE(Xia and Xie 2001).

Reference

Chan PP, Lowe TM (2019) tRNAscan-SE: Searching for tRNA Genes in Genomic Sequences. In: Kollmar M (ed) Gene Prediction: Methods and Protocols. Springer New York, New York, NY, pp 1-14

Kück P, Meid SA, Groß C, Wägele JW, Misof B (2014) AliGROOVE – visualization of heterogeneous sequence divergence within multiple sequence alignments and detection of inflated branch support. BMC Bioinformatics 15(1):294 doi:10.1186/1471-2105-15-294

Xia X, Xie Z (2001) DAMBE: Software Package for Data Analysis in Molecular Biology and Evolution. Journal of Heredity 92(4):371-373 doi:10.1093/jhered/92.4.371
